# Single-cell RNA sequencing reveals clonally expanded CD4^+^ tissue-resident memory T cells in histidyl-tRNA synthetase–induced myositis

**DOI:** 10.1172/jci.insight.208009

**Published:** 2026-07-23

**Authors:** Decheng Li, Daniel P. Reay, Iago Pinal-Fernandez, Maria Casal-Dominguez, Andrew L. Mammen, Sarah L. Gaffen, Timothy B. Oriss, Dana P. Ascherman

**Affiliations:** 1 Division of Rheumatology and Clinical Immunology, Department of Medicine, University of Pittsburgh, Pittsburgh, Pennsylvania, USA.; 2 Tsinghua Medicine, Tsinghua University, Beijing, China.; 3 National Institute of Arthritis and Musculoskeletal and Skin Diseases, National Institutes of Health, Bethesda, Maryland, USA.; 4 Department of Neurology and; 5 Department of Medicine, Johns Hopkins University School of Medicine, Baltimore, Maryland, USA.

**Keywords:** Autoimmunity, Immunology, Muscle, T cells, Transcriptomics

## Abstract

The precise mechanisms underlying the pathogenesis of idiopathic inflammatory myopathy (IIM) remain undefined. However, there has been increasing recognition that tissue-resident memory T cells (TRMs) play an important role in the pathogenesis of systemic autoimmune disease. In IIM, TRM-associated transcriptional signatures have been reported but on a very limited basis. By using multimodal single-cell RNA-sequencing analysis in our established murine model of histidyl-tRNA synthetase–induced myositis, we identified a prominent population of CD4^+^ TRMs in inflamed skeletal muscle. Muscle CD4^+^ TRMs exhibited high expression of genes encoding *Cd69*, *Cxcr6*, *Runx3*, and *Prdm1*, alongside low expression of *Klf2*, *Ccr7*, *Sell*, *S1pr1*, and *Tcf7* — a profile that is generally consistent with previous reports of TRM gene signature and that we validate through comparison with transcriptomic profiles of human muscle tissue. Detailed pathway analysis in our model indicates that muscle CD4^+^ TRMs contribute to innate immune regulatory pathways enriched for TNF and IFN-γ signaling. Furthermore, analysis of TCR clonotype distribution and CDR3 sequence similarity revealed pronounced clonal expansion of CD4^+^ TRMs relative to other T cell subsets — a pattern that remained stable from 2 to 6 weeks after immunization. Collectively, these results suggest a potential role for CD4^+^ TRMs in the pathogenesis of autoimmune myositis.

## Abstract 2

This work illustrates the transcriptional profile of resident memory T cells in a murine model of HRS-induced myositis, replicating transcriptional signatures found in human disease.

## Introduction

Idiopathic inflammatory myopathies (IIMs) are autoimmune disorders characterized by skeletal muscle dysfunction as well as extramuscular manifestations that include characteristic skin rashes, Raynaud’s phenomenon, inflammatory arthritis, and interstitial lung disease (1–3). Because of the heterogeneity among IIM subtypes and incomplete understanding of their pathogenic mechanisms, therapeutic intervention in IIM is still relatively limited in scope to global immunosuppressive agents — highlighting the need to further elucidate immune pathways such as those associated with tissue memory.

Histidyl-tRNA synthetase (HRS; also known as Jo-1) represents one of the most common autoantigens in human IIM and, along with several other less commonly targeted tRNA synthetases, marks the clinical subset known as the anti-synthetase syndrome. To investigate disease mechanisms, we employed our well-established mouse model of HRS-induced myositis, which replicates many key features of human IIM (4). Prior studies in this model have shown that innate immune activation promotes T cell infiltration and that antigen-specific CD4^+^ T cells are required for persistent muscle inflammation (5). However, the role of individual T cell subsets and cytokine profiles within inflamed muscle tissues has not yet been fully demonstrated.

Among T cell subsets, tissue-resident memory T cells (TRMs) have garnered considerable attention in recent years as key mediators of tissue pathology in chronic inflammatory and multiorgan autoimmune diseases. TRMs constitute a population of T lymphocytes that reside in peripheral tissues with limited circulation via either the bloodstream or lymphatics (6). Although they have been identified in numerous anatomical sites, TRMs are primarily located in barrier tissues such as mucosal surfaces and, as such, are unlike circulating central memory T cells (TCMs) or effector memory T cells (TEMs), which do have the ability to reenter blood or lymphoid organs.

Despite thorough characterization of CD8^+^ TRMs, CD4^+^ TRMs remain comparatively understudied. A major challenge lies in their pronounced heterogeneity across tissues and diseases, hindering the ability to characterize a common CD4^+^ TRM precursor (7). Nevertheless, compelling evidence for CD4^+^ TRMs has been reported in the lung (8), skin (9), female reproductive tract (10), small intestine (11), bone marrow (12), and kidney (13, 14). Canonical TRM-associated markers include CD69 and CD103, both of which are involved in promoting tissue retention. However, while CD69 is widely expressed on CD4^+^ TRMs, CD103 appears to be far less prominently expressed in this subset (9, 15–17). Similarly, the chemokine receptor CXCR6 also plays a critical role in TRM tissue localization (18–20). On the other hand, CD62L and CCR7, which are associated with cell circulation, are absent in CD4^+^ TRMs, preventing these cells from reentering the bloodstream or lymphatics (21). Beyond cell surface proteins, a number of transcription factors, such as HOBIT, BLIMP-1 (22), and RUNX3 (23), have been implicated in regulating TRM development and function, highlighting the fact that overall transcriptional profile may be more reliably descriptive of TRM phenotype than expression of cell surface markers alone.

Typically, TRMs permanently reside within non-lymphoid tissues. Upon antigen reexposure, or in some cases as a nonspecific response to inflammatory stimuli, TRMs orchestrate rapid immune responses and enhance pathogen clearance (24, 25). In addition to direct antigen-specific activity, TRMs also interact with other innate and adaptive immune cells, such as macrophages (26, 27), regulatory T cells (Tregs) (28, 29), and neutrophils (30). Recent studies further highlight their potential involvement in inflammatory and autoimmune diseases (31, 32). Owing to their long-lived tissue residency, TRMs have the opportunity to repeatedly encounter autoantigens, which can lead to recurrent destructive inflammation, as observed in vitiligo, psoriasis, and cutaneous lupus erythematosus (33).

In the case of human IIM, single-cell profiling of inflamed muscle has revealed TRM-associated transcriptional signatures across several disease subsets — albeit in a single study limited to 7 specimens (34). Through detailed analysis of an unparalleled repository of human IIM by bulk RNA sequencing, we now demonstrate enrichment of TRM-associated transcriptional signatures in inflamed muscle. However, the function and importance of TRMs in human IIM remain unclear, either through mechanistic or computational analyses. Furthermore, no current murine model of myositis has identified the presence of TRMs, thus hindering the mechanistic dissection of their development and contribution to myositis.

Motivated by these gaps in understanding the biology of TRMs in IIM, this study was designed to comprehensively characterize the transcriptomic landscape of CD4^+^ TRMs in our well-established model of HRS-induced myositis, to define their potential role in disease pathogenesis, and to suggest targetable pathways for therapeutic intervention. These studies demonstrate, for the first time to our knowledge, the presence of a prominent IFN-γ–producing subset of CD4^+^ TRMs in murine muscle tissue that is consistent with transcriptional signatures derived from bulk RNA sequencing of human muscle specimens, validating our model as an important tool to understand the pathogenic role of TRMs in human IIM.

## Results

### Upregulation of signature genes associated with TRMs and type II interferon signaling in human anti–Jo-1 myositis.

**Figure 1:**
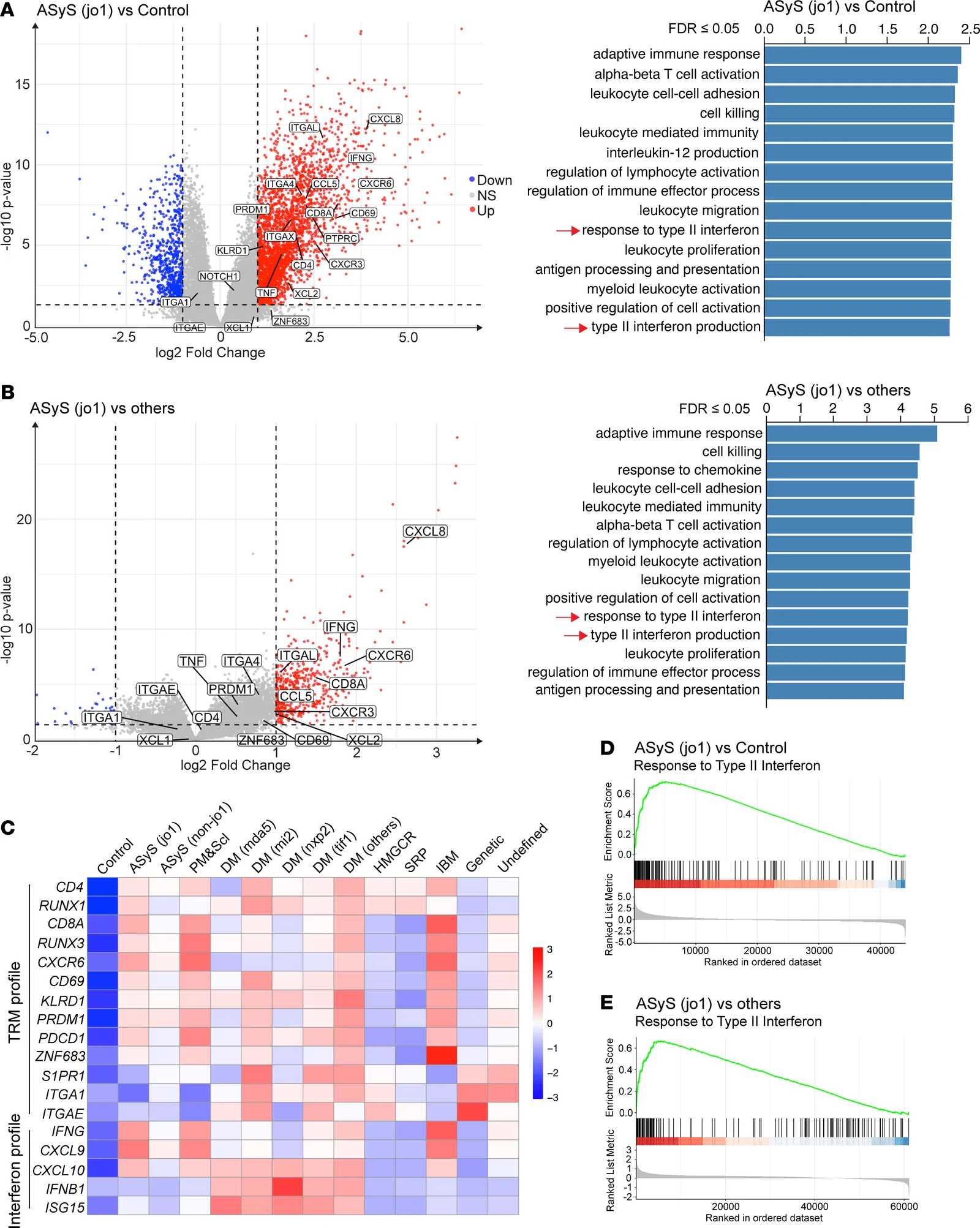
Bulk RNA sequencing analysis of human myositis biopsy specimens. (**A**) Left: Volcano plot displaying differentially expressed genes (DEGs) in anti–Jo-1 myositis patients (*n* = 37) compared with healthy controls (*n* = 37), identified by Wilcoxon’s rank-sum test, with adjusted *P* value < 0.05 and log_2_ fold change (FC) > 1 or < –1. Genes with log_2_ FC > 1 and adjusted *P* < 0.05 were designated as upregulated (Up); those with a log_2_ FC < –1 and adjusted *P* < 0.05 were classified as downregulated (Down). Right: Bar plot demonstrating Gene Ontology (GO) enrichment analysis comparing anti–Jo-1 myositis with healthy muscle, ordered by normalized enrichment scores. FDR < 0.05; minimum gene IDs in category = 20. (**B**) Left: Volcano plot displaying DEGs in anti–Jo-1 myositis patients (*n* = 37) compared with all other myositis subtypes (*n* = 625). Right: Bar plot demonstrating GO enrichment analysis ordered by normalized enrichment scores. FDR < 0.05; minimum gene IDs in category = 20. (**C**) The depicted heatmap demonstrates relative expression of key genes representative of resident memory T cells (TRMs) in different subsets of inflammatory myopathy, genetic myopathies, and healthy controls. Genes reflecting type I versus type II interferon responses are included. (**D** and **E**) Illustration of gene set enrichment for selected biological processes identified by GO comparing anti–Jo-1 myositis with non-myositis controls (**D**) or other subtypes of myositis (**E**). ASyS, anti-synthetase syndrome; DM, dermatomyositis; IBM, inclusion body myositis; PM, polymyositis; Scl, scleroderma; HMGCR, HMG-CoA reductase (3-hydroxy-3-methylglutaryl-CoA reductase); SRP, signal recognition particle.

Given the important role of TRMs in driving autoimmune disease responses (33, 35–37), we used bulk RNA sequencing to assess a large compendium of muscle biopsy specimens (*n* = 662) derived from patients with various IIM subtypes and other non-inflammatory muscle conditions for transcriptional evidence of TRMs. Consistent with prior findings, genes encoding TRM-associated cell surface markers and adhesion molecules were significantly upregulated in muscle tissue of the anti–Jo-1 myositis subset compared with muscle tissue from healthy controls, including *CD69*, *CXCR6*, and *CXCR3* (Figure 1A). Additionally, TRM-related transcription factors, such as *ZNF683* and *PRDM1*, also exhibited increased expression in diseased muscle tissue from anti–Jo-1 myositis. Notably, expression of many TRM signature genes appeared to be preferentially upregulated in anti–Jo-1 myositis relative to other myositis subtypes (Figure 1B). When compared with subsets of dermatomyositis (DM), immune-mediated necrotizing myopathy (IMNM), and inclusion body myositis (IBM), for example, anti–Jo-1 myositis tissue demonstrated significantly elevated levels of *ITGAL*, *CXCR6*, and *CXCR3*, further indicating a prominent tissue-resident T cell phenotype. As shown by additional volcano plots comparing anti–Jo-1 myositis with different subsets of myositis (Supplemental Figure 1, A–C; supplemental material available online with this article; https://doi.org/10.1172/jci.insight.208009DS1) and the heatmap of Figure 1C, the profile of TRM signature genes clearly distinguished anti–Jo-1 myositis from non–Jo-1 anti-synthetase myositis, DM, and IMNM — but overlapped considerably with anti-PM/Scl and IBM subsets.

Beyond these specific TRM-associated genes/transcription factors, enrichment analysis based on Gene Ontology (GO) revealed several immune regulatory programs potentially involved in the pathogenesis of anti–Jo-1 myositis (Figure 1, A and B). Chief among these were transcriptional programs associated with antigen recognition, adaptive immune responses, and type II interferon (IFN-γ) signaling. Notably, gene set enrichment analysis (GSEA) demonstrated that both the production of, and the response to, type II interferon were increased, suggesting a potential regulatory network driven by IFN-γ signaling in anti–Jo-1 myositis (Figure 1, D and E; and Supplemental Figure 1D) distinct from that of non–Jo-1 anti-synthetase and DM subsets (the latter dominated by type I interferon; Supplemental Figure 1E).

Given these results, we sought to determine whether a similar transcriptional profile could be identified in our murine model of HRS-induced myositis. Bulk RNA sequencing of hamstring muscle tissue revealed that the murine model consistently exhibited a comparable gene expression pattern, with high expression of *Ifng*, *Cd69*, *Cxcr6*, and *Prdm1*, as well as low expression of *Cd103* (Supplemental Figure 2A). Importantly, enrichment of genes linked to adaptive immunity and type II interferon signaling was also detected in muscle tissue from the mouse model (5) (Supplemental Figure 2B), further paralleling human disease.

### scRNA-seq profiles of muscle-infiltrating cells in experimental HRS-induced myositis reveals the presence of CD4^+^ tissue-resident memory cells.

**Figure 2:**
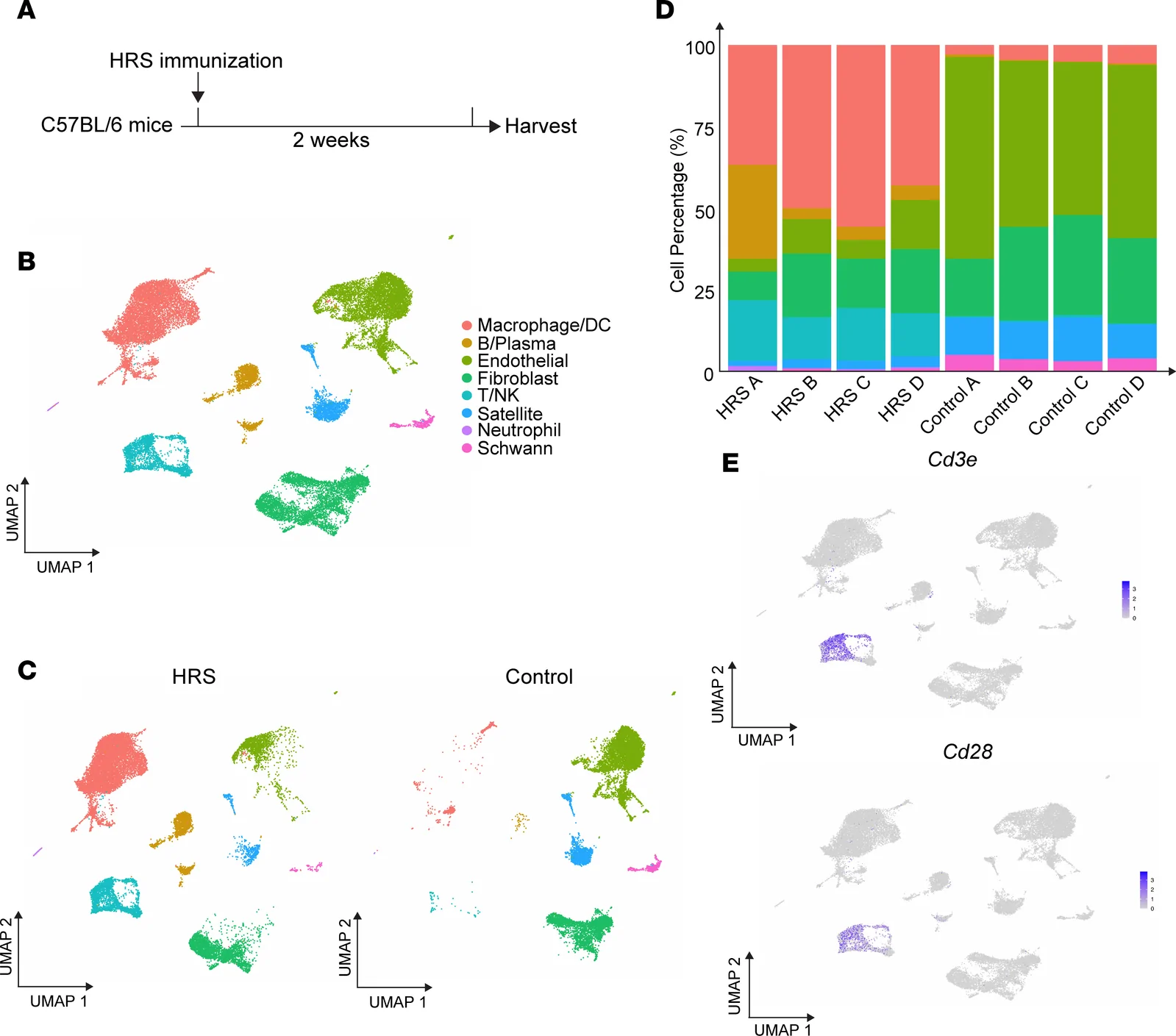
Single-cell profile in murine muscle with histidyl-tRNA synthetase–induced myositis. (**A**) Histidyl-tRNA synthetase (HRS) immunization protocol for induction of experimental murine myositis. (**B**) UMAP visualization of 8 clusters identified in muscle-infiltrating cells of mice with HRS-induced myositis, 2 weeks after immunization (*n* = 28,903 cells). (**C**) UMAP visualization of 8 clusters split into 2 groups corresponding to HRS-immunized versus PBS control mice. While HRS group A and PBS control group A each consisted of pooled muscle cell–infiltrating cells from 2 mice, HRS groups B–D and PBS control groups B–D each consisted of pooled cells from 3–5 mice. (**D**) Percentage of each cell cluster in total cell counts across different sample groups (HRS A–D, PBS A–D). (**E**) The normalized expression of *Cd3e* and *Cd28* visualized in feature plots.

Based on these observations derived from bulk RNA sequencing of muscle tissue from human IIM subsets as well as our mouse model, we next performed scRNA-seq of hamstring muscle tissue in HRS-induced myositis to more precisely characterize the gene expression profile of muscle-infiltrating TRMs. Per established protocol, C57BL/6 mice were immunized with a single dose of recombinant murine HRS (diluted in PBS) in bilateral hamstring muscles, a procedure that uniformly induces robust in situ inflammation (5, 38, 39). Two weeks after immunization (a time point corresponding to peak inflammation) (39), muscle-infiltrating and other tissue-resident cells were isolated and processed to allow for RNA extraction, library preparation, and subsequent scRNA-seq (Figure 2A). Samples from individual mice were pooled as outlined in Figure 2, and datasets from 4 independent experiments were analyzed simultaneously.

After quality control, batch correction, and normalization, 28,903 cells were obtained for analysis. Unsupervised clustering of these cells was performed, which identified 8 clusters (annotated by known genes) that were then visualized by uniform manifold approximation and projection (UMAP) (Figure 2B and Supplemental Figure 3). In comparison with the PBS-treated control group, HRS-immunized mice demonstrated significant infiltration of immune cells, including macrophages/dendritic cells (DCs), B cells, and T/NK cells (Figure 2C). Notably, neutrophils represented only a small percentage of cells isolated from muscle tissue of mice immunized with recombinant HRS (Figure 2D).

**Figure 3:**
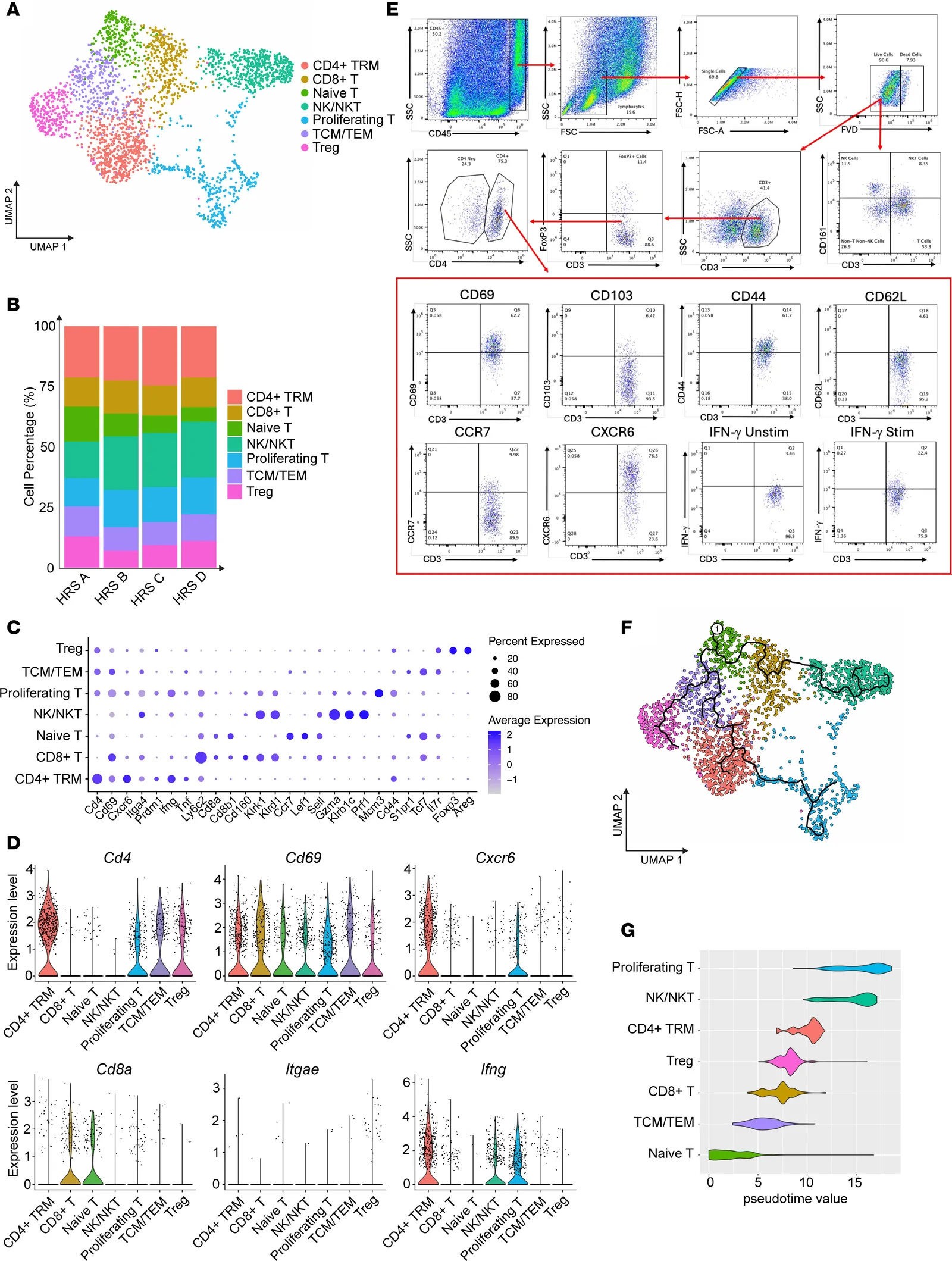
T/NK cell subclusters in muscle tissue from HRS-induced myositis. (**A**) UMAP visualization of subsets of T/NK cells isolated from muscle tissue in HRS-induced myositis. (**B**) Percentage of each subcluster in total T/NK cell counts displayed across different samples. (**C**) Dot plot displaying the expression level of highly expressed genes for each subcluster. (**D**) Normalized expression of *Cd4*, *Cd8a*, *Cd69*, *Cd103*, *Cxcr6*, and *Ifng* visualized in violin plots. (**E**) Cells isolated from muscle were analyzed by spectral flow cytometry. Data show CD3^+^CD4^+^, CD3^+^FoxP3^+^, and CD3^+^CD161^+^ cells; more detailed assessment of CD3^+^CD4^+^CD69^+^ cells demonstrates a CD44^+^CD62L^lo^CCR7^lo^CXCR6^hi^ phenotype. Also shown is IFN-γ expression with or without in vitro stimulation with αTCR activation beads and PMA/ionomycin. (**F**) Pseudotime trajectory combined with identified cell clusters in T/NK subsets, visualized in the depicted UMAP. Color corresponds to the clusters shown in **A**. (**G**) Pseudotime values across different T/NK subsets, visualized via violin plots and aligned from low to high.

The cluster containing T and NK cells was initially annotated based on the top 10 marker genes within this cluster (Supplemental Figure 4A), including *Cd3e* and *Cd28*, both of which were found exclusively in this cluster on the UMAP (Figure 2E). To analyze the T cell profile of HRS-immunized mice in greater detail, the T/NK cluster was separated and renormalized for subclustering. We recovered 2,698 cells from this analysis, leading to the identification of 7 clusters within the overall T/NK population as visualized by UMAP (Figure 3A). Notably, NK and NKT cells were clearly delineated from T cell subpopulations following this reclustering process. As in the initial analysis, each subcluster was identified based on its top 10 marker genes (Supplemental Figure 4B). Using annotations derived from known resident memory marker genes (40, 41), this analysis revealed that, on average, CD4^+^ TRMs accounted for 22% of the original T/NK cell cluster (Figure 3B).

We then assessed levels of gene expression for previously described TRM markers representing cell surface as well as intracellular molecules — including *Cd69*, *Cd103*, *Itgal*, *Cxcr3*, *Cd44*, *Ifng*, and several different transcription factors (40–42) (Supplemental Table 1). In addition to being a general marker of T cell activation, the protein encoded by *Cd69* inhibits T cell egress from resident tissues and is one of the most prominent markers of CD4^+^ TRMs. *Cd69* was widely expressed across multiple T cell clusters and is therefore not exclusive to CD4^+^ TRMs (Figure 3, C and D). Interestingly, another adhesion molecule commonly associated with TRMs, *Cd103* (*Itgae*), was not highly expressed by the CD4^+^ TRMs isolated from involved muscle (Figure 3D). An additional adhesion molecule encoded by *Itgal* (CD11a) (8) was expressed across a range of cells, but appeared to be mostly concentrated within CD4^+^ TRMs as well as NK/NKT cells (Supplemental Figure 5, A and B). The chemokine receptor CXCR6 has been widely reported as a major marker of TRMs residing in various tissues (18–20), which was corroborated by the highly enriched expression of *Cxcr6* in the CD4^+^ TRM cluster. *Cxcr3* expression followed a similar pattern across cell clusters (43) (Supplemental Figure 5, A and B). Although not limited to CD4^+^ TRMs, expression of the T cell activation marker gene *Cd44* was nonetheless elevated, consistent with a memory-like signature (Figure 3C). *Ifng* was also expressed in CD4^+^ TRMs and, to a lesser extent, NK and NKT cells (Figure 3, C and D). Intriguingly, *Ifng* expression was found in both proliferating and non-proliferating CD4^+^ TRMs and CD8^+^ T cells (Figure 3D).

Of the transcription factors commonly associated with TRMs, *Prdm1* (Blimp-1) expression and *Notch1* (Notch) expression were both increased in CD4^+^ TRMs (Figure 3C), while *Zfp683* (Hobit) was not (22, 44) (Supplemental Figure 5A). *Runx3* was expressed in CD4^+^ TRMs as expected but was also observed in CD8^+^ T cells as well as other subsets of NK/NKT cells (Supplemental Figure 5A). Several other genes reported to be decreased in TRMs, including *S1pr1*, *Sell* (CD62L), and *Tcf7* (TCF1) (21), were, in fact, expressed at low levels in CD4^+^ TRMs relative to other cells in the T/NK cluster (Figure 3C). Thus, these transcription profiles were consistent with a tissue-resident memory CD4^+^ T cell phenotype residing in inflamed muscle.

Among other cell types within the original T/NK cluster, CD8^+^ T cells and NK/NKT cells shared some cytotoxicity-related genes (*Cd7* [ref. 45], *Klrd1*, *Klrk1*) that were distinct from the expression of NK-specific marker genes such as *Gama* and *Prf1* (46). Proliferating T cells uniquely expressed cell cycle regulation genes such as *Pclaf* and *Mcm3* but also shared a number of marker genes with CD4^+^ TRMs and CD8^+^ T cells (e.g., *Cd44*, *Cxcr6*, *Cxcr3*, *Ifng*, *Prdm1*, *Runx3*, *Notch1*) (Supplemental Figure 5, A and B). The cluster containing central memory T cells (TCMs) and effector memory T cells (TEMs) was delineated based on high expression of *Il7r*, *Tcf7*, and *Slamf6* (47). Importantly, the gene expression signature of murine TRMs from scRNA-seq analysis paralleled the bulk RNA-sequencing analysis of human myositis muscle tissue with regard to key markers. Coupled with Flex scRNA-seq of archived human muscle biopsy specimens demonstrating TRM expression profiles in muscle-infiltrating CD4^+^ T cells linked to different subsets of myositis (including anti–Jo-1 myositis; Supplemental Figures 6 and 7), these results provide compelling evidence that CD4^+^ TRMs are associated with human anti–Jo-1 myositis and thus could be a critical area for future investigation.

In order to assess TRM phenotype at the protein level, we performed flow cytometry analysis of muscle-infiltrating/resident cells. Various T cell subsets, including FoxP3^+^ Tregs, as well as NK and NKT cells, could be readily discerned (Figure 3E). The relative proportions of each were generally comparable to those obtained by sequencing (Figure 3B). The CD4^+^ TRM cells were very similar to the scRNA-seq subset in terms of high CD69 expression with comparatively low CD103. This analysis aligned with the expected activation phenotype of CD4^+^CD69^+^ cells indicated by RNA sequencing (Figure 3, C and D), demonstrating the following cell surface expression profile: CD44^+^CD62L^lo^CCR7^lo^CXCR6^hi^. Importantly, these cells expressed IFN-γ following in vitro reactivation (Figure 3E). More detailed analysis demonstrated that CD69^+^CCR7^–^CXCR6^hi^-gated cells (marking CD4^+^ TRMs) were capable of producing IFN-γ (see below), consistent with findings from our transcriptomic analysis.

The scRNA-seq subclustering analysis did not definitively identify CD8^+^ TRMs (Figure 3A), and flow analysis also failed to support a significant population of CD8^+^ TRMs based on relatively low CD44 expression combined with relatively high expression of CD62L and CCR7 (Supplemental Figure 8). Notably, FoxP3^+^ Tregs presented with a cell surface phenotype distinct from other CD4^+^ and CD8^+^ T cells (Supplemental Figure 8), consistent with findings at the level of RNA expression (Figure 3, C and D). Therefore, although direct quantitative comparison between the two methods was limited by variations in the respective phenotypes identified, the relative proportions of muscle-infiltrating inflammatory cells and their overall phenotype were in general agreement between scRNA-seq and flow cytometric approaches.

To investigate the temporal/developmental relationship between different cell types represented in the T/NK cell cluster, we performed pseudotime analysis. Using naive T cells as a point of origin, this analysis revealed two major trajectories in T/NK subsets. One branching trajectory corresponded to CD8^+^ T cells, and the other corresponded to CD4^+^ T cells and Tregs (Figure 3F). These trajectories highlighted the distinctive developmental pathway/branch points of CD4^+^ TRMs within the CD4^+^ lineage. In addition, the pseudotime value of each cell subset suggested that CD4^+^ TRMs occupy a later developmental stage compared with TCMs/TEMs and Tregs (Figure 3G). Overall, this pseudotime analysis indicated a possible differentiation progressing from naive T cells to TCMs/TEMs to CD4^+^ TRMs (48).

### Pathway and cell-cell communication analysis indicates participation of CD4^+^ TRMs in myositis-associated immune responses.

**Figure 4:**
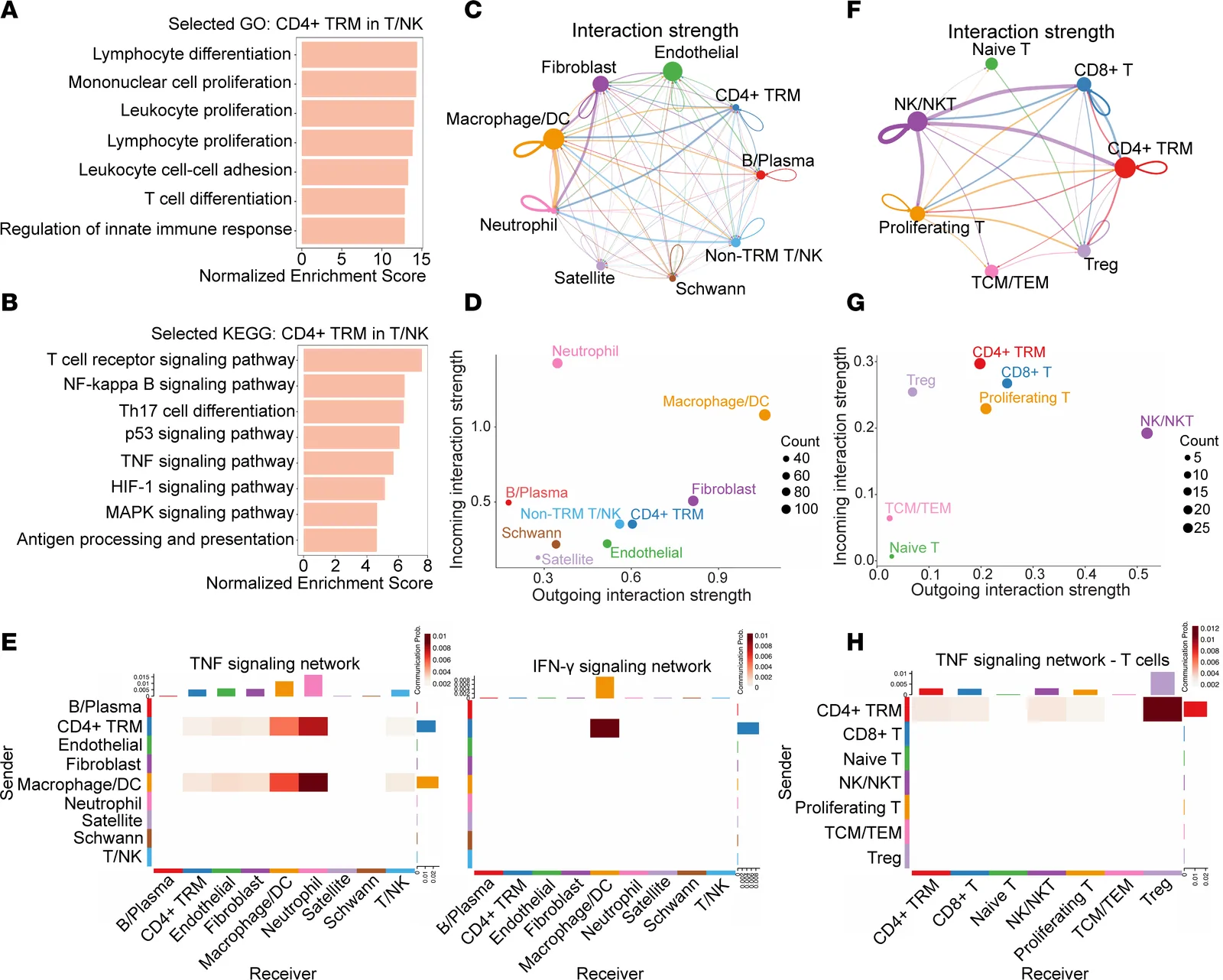
Functional characterization of TRMs and interactions between TRMs and other cells. (**A** and **B**) Pathway analysis based on DEGs of CD4^+^ TRMs in T/NK subsets. Pathways generated from GO were selected from the top 40 pathways identified (**A**), and pathways generated from KEGG were selected from the top 70 (**B**). (**C** and **D**) Interaction strength between CD4^+^ TRMs and all clusters, visualized by network and scatter plots. Interaction strength represents ligand-receptor–mediated intercellular communication probability, measured by CellChat. Vertex size is proportional to cell counts in each cluster (**C**). In scatter plots displaying sources and targets across all clusters (**D**), dot size is proportional to the number of inferred outgoing and incoming links associated with each cell cluster. (**E**) Heatmap visualizing TNF and IFN-γ signaling networks across different clusters. Horizontal and longitudinal axes indicate incoming and outgoing signals, respectively. (**F** and **G**) Interaction strength between CD4^+^ TRMs and other subsets of T/NK cells, again depicted as network and scatter plots with parameters as described for plots shown in **C** and **D**. (**H**) Heatmap visualizing TNF signaling network across different subsets within T/NK cells. Horizontal and longitudinal axes indicate respective incoming and outgoing signals.

Despite numerous investigations of CD8^+^ TRMs in recent years, characterization of CD4^+^ TRMs has not been as thoroughly elucidated (7). In order to gain insight into the functional significance of CD4^+^ TRMs in our model of HRS-induced myositis, we conducted GO and Kyoto Encyclopedia of Genes and Genomes (KEGG) enrichment analysis on differentially expressed genes in CD4^+^ TRMs within the T/NK cluster of muscle-infiltrating lymphocytes (Figure 4, A and B). Pathways were filtered based on an adjusted *P* value less than 0.05 and sorted by normalized enrichment score, from high to low, after which the top 70 relevant pathways were selected (Supplemental Tables 2 and 3). These analyses indicated that CD4^+^ TRMs participate in immune cell differentiation and proliferation, as well as regulation of different aspects of the innate immune response. With regard to signaling pathways, TCR activation was notable in CD4^+^ TRMs as expected, as were pathways associated with downstream activation of NF-κB, TNF, and MAPK.

To delve deeper into signaling pathways mediated by CD4^+^ TRMs, we performed cell-cell communication analysis with CellChat, a software algorithm based on predicted ligand-receptor pairs. This analysis demonstrated that CD4^+^ TRMs exhibited distinct interaction characteristics that were unique in comparison with other cell subsets. Review of Figure 4 indicates that muscle-residing CD4^+^ TRMs showed strong interactions with macrophages/DCs and neutrophils (Figure 4, C and D), despite the fact that neutrophils represent a small percentage of muscle-infiltrating cell populations based on histology, flow cytometry, and cell clustering analyses (which could be skewed by neutrophil fragmentation and/or low mRNA content). When interaction strength was assessed, CD4^+^ TRMs demonstrated slightly higher outgoing strength, but comparable incoming strength, relative to the other cell types (Figure 4, C and D). Among the signaling interactions observed in muscle-infiltrating cells (Supplemental Figure 9), TNF- and IFN-γ–mediated signals were notably transduced from CD4^+^ TRMs to macrophages or DCs (Figure 4E). Migration inhibitory factor (MIF), a proinflammatory chemokine regulating macrophage migration, was also expressed by CD4^+^ TRMs. These findings suggested a robust reciprocal communication between CD4^+^ TRMs and macrophages (as well as DCs) in HRS-induced myositis, aligning with previous reports that chemokines from macrophages are involved in sustaining TRMs (49, 50), which, in turn, are important for macrophage tissue retention (51).

Beyond these reciprocal interactions with macrophages, we investigated communication networks between CD4^+^ TRMs and other muscle-infiltrating T cell populations (Figure 4, F and G, and Supplemental Figure 10). These analyses demonstrated that although CD4^+^ TRMs delivered signals to CD8^+^ T cells, proliferating T cells, and Tregs, incoming signals from other cell types were more prominent (Figure 4G). One of the most notable observed interactions involved the TNF/TNFR2 signaling network, which showed TNF-mediated signals delivered from CD4^+^ TRMs to Tregs (Figure 4H). This observation is of potential importance since TNFR2 (*Tnfsfr1*) expression is largely responsible for maximizing the suppressive function of Tregs (52, 53), suggesting a dual, possibly inflammation-balancing, role for CD4^+^ TRMs in both promotion of muscle pathology and activation of suppressive Treg function.

### Clonally expanded CD4^+^ TRMs predominate in HRS-induced myositis.

**Figure 5:**
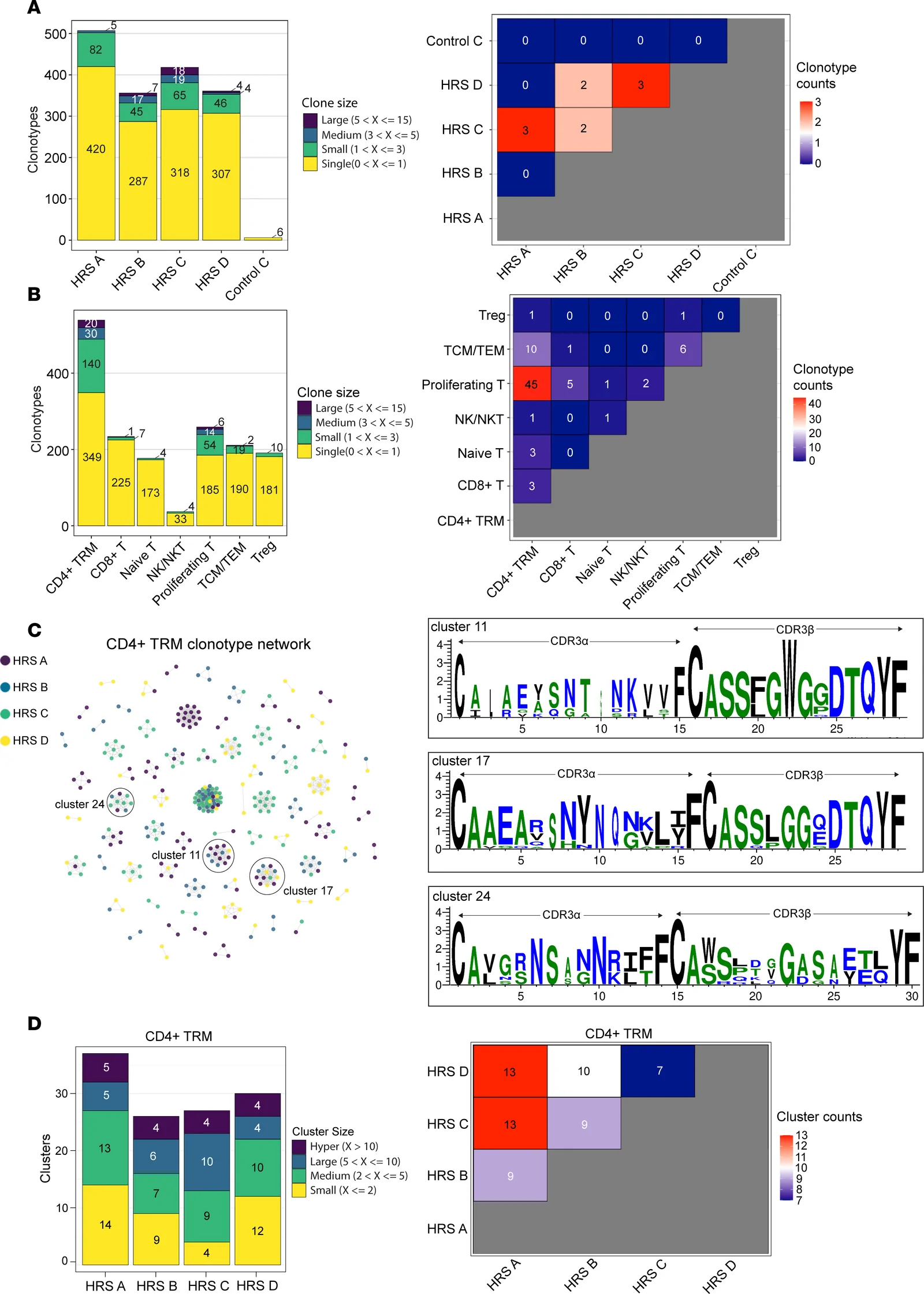
Expanded clonotypes are identified in muscle tissue from HRS-induced myositis. (**A**) While the bar graphs demonstrate counts of T cell clonotypes (cell counts from a specific clonotype) across 5 independent experimental groups of mice (*n* = 4 HRS-immunized, *n* = 1 PBS control group), the checkerboard matrix depicts the number of overlapping clonotypes as a heatmap. Clonotypes were divided into 4 groups based on the number of identical TCR clones (5 < large ≤ 15; 3 < medium ≤ 5; 1 < small ≤ 3; single = 1). (**B**) Complementary analyses show clonotype counts across 7 subsets of T/NK cells (bar graphs), with the number of overlapping clonotypes illustrated by the accompanying heatmap. (**C**) Clustering network of different clonotypes from CD4^+^ TRMs with high sequence similarity, where each dot represents a specific clonotype and cluster size indicates the number of connections with different clonotypes. The accompanying sequence logo plot shows CDR3 (TCRα and TCRβ) sequence alignments of 3 clusters of clonotypes selected from this plot (circled and numbered). (**D**) Clusters of closely related clonotypes with a high degree of similarity in TCR sequence were divided into 4 groups based on cell counts contained within the specified cluster (hyper > 10; 5 < large < 10; 2 < medium ≤ 5; small ≤ 2). Right: Counts of shared clusters across 4 HRS-treated groups, with the number of overlapping clusters represented by the accompanying heatmap.

By employing TCR sequencing in combination with scRNA-seq, we comprehensively analyzed clonotypes in different T cell subsets across multiple independent experiments. These analyses provided insights regarding the clonal expansion of CD4^+^ TRMs as well as the activation status of T cells within inflamed muscle. Clonal expansion was classified as large (5 < *x* ≤ 15), medium (3 < *x* ≤ 5), and small (1 < *x* ≤ 3) based on the number of expanded clones sharing identical TCRα/TCRβ CDR3 sequences. Note that none of the clonotypes were represented by more than 15 cells. Importantly, the expansion levels were similar in 4 independent experiments, with control groups showing no T cell clonal expansion (Figure 5A and Supplemental Table 4). Among all muscle-infiltrating cell types within the T/NK cell cluster, CD4^+^ TRMs and proliferating T cells exhibited the highest frequency of expanded clonotypes. In CD4^+^ TRMs, for example, we detected a total of 20 expanded clonotypes containing between 5 and 15 cells (Figure 5B).

Shared clonotypes were evaluated between different cell populations within the T/NK cell cluster in 4 independent experiments. Each experiment shared 3 to 8 clonotypes with the other independent experiments, strongly suggesting a highly reproducible antigen-specific T cell response in HRS-induced myositis. Intriguingly, several clonotypes were shared between CD4^+^ TRMs and proliferating T cells, demonstrating that a portion of CD4^+^ TRMs were undergoing a proliferative process indicative of ongoing TRM activation (Figure 5B and Supplemental Table 5).

Based on previously published research, the antigen specificity of TCRs is highly dependent on a short protein motif in the CDR3 sequence (54). Because CDR3 sequences demonstrating sequence homology indicate shared processes involved in antigen recognition and clonotype convergence (54–57), we analyzed the similarity of TCRα/TCRβ CDR3 amino acid sequences within CD4^+^ TRM subsets derived from 4 independent experiments. An algorithm named igraph (https://igraph.org/) was used to group closely related clonotypes into clusters (Figure 5C and Supplemental Tables 6 and 7). Clusters 11, 17, and 24 were each composed of related clonotypes from different experimental samples demonstrating a high degree of TCRα/TCRβ CDR3 sequence consensus, with a similar start sequence of CASS/CASG (Figure 5C). Further analysis showed that many of the remaining clusters were also composed of clonotypes from separate experiments (Figure 5D). Notably, the presence of clusters composed of clonotypes from multiple samples across independent datasets strongly suggested shared antigen recognition (likely driven by HRS) based on similarity of TCR sequences (Figure 5, C and D).

### Refinement of CD4^+^ TRM gene signature and function during long-term follow-up of HRS-induced myositis.

**Figure 6:**
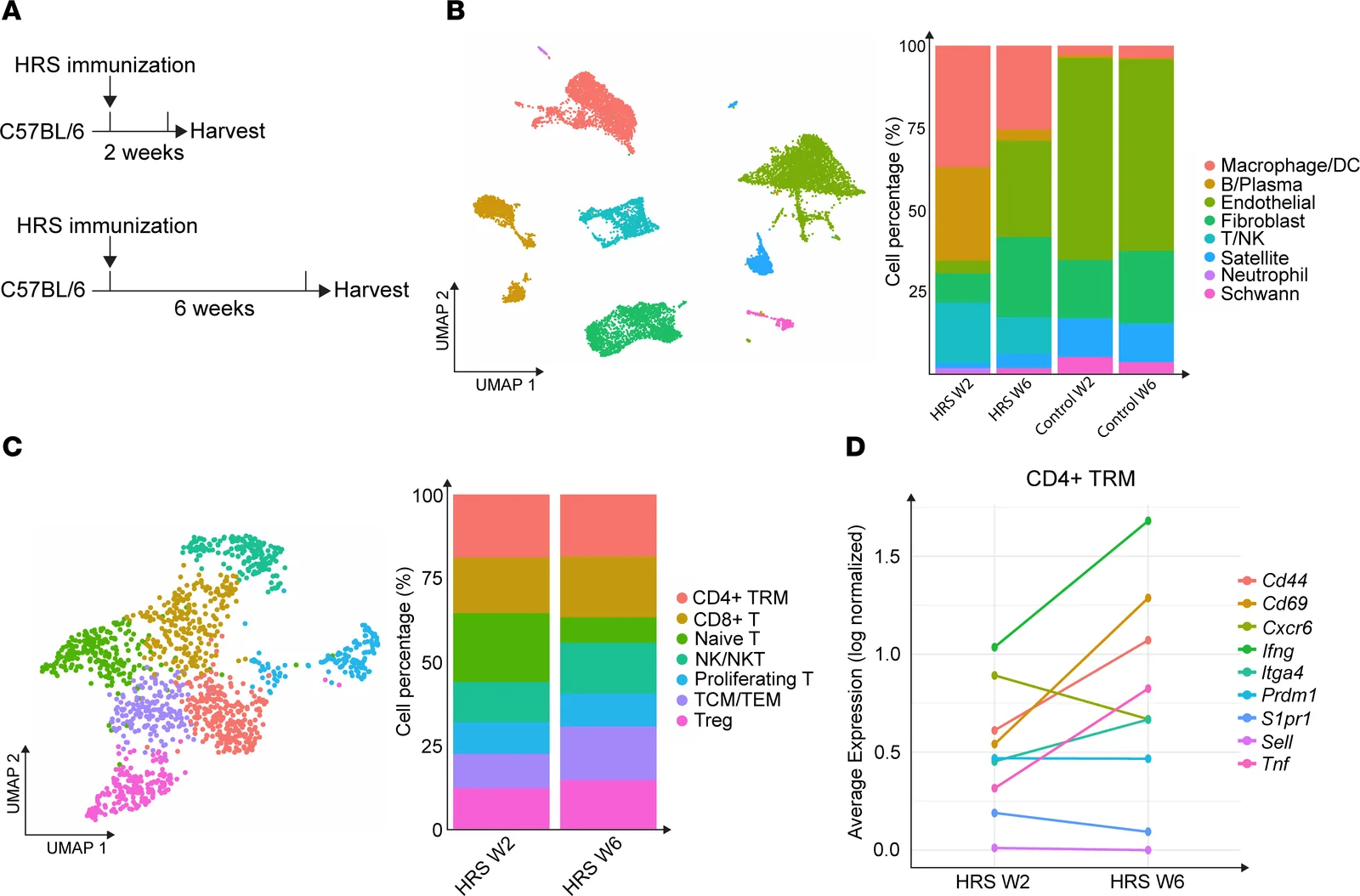
CD4^+^ TRM profile is maintained from 2 to 6 weeks after HRS immunization. (**A**) HRS immunization protocol for assessment of experimental murine myositis at different time points. (**B**) UMAP visualization of 8 clusters identified in muscle-infiltrating cells from mice with HRS-induced myositis, with the percentage of each cell cluster relative to total cell number shown in the bar graphs at right. Data were pooled from mice immunized with recombinant HRS at 2 weeks (*n* = 2) and 6 weeks (*n* = 3) after immunization. (**C**) UMAP visualization of T/NK cell subclusters, along with the percentage of each subcluster of T/NK cells (bar graphs, right). (**D**) Average expression level of signature genes in CD4^+^ TRMs at 2 versus 6 weeks after immunization.

To assess the profile of CD4^+^ TRMs over time, we performed scRNA-seq analysis of muscle tissue at a later time point following immunization with HRS, namely at 6 weeks after immunization (Figure 6A). Muscle-infiltrating cells were processed for scRNA-seq as described above. After quality control and normalization, 13,598 cells were recovered for analysis. Subsequently, unsupervised clustering of muscle-infiltrating cells was conducted using the same standard as in the previously presented analysis of muscle tissue harvested 2 weeks after immunization. Again, 8 clusters annotated by designated marker genes were identified and visualized by UMAP (Figure 6B). Comparison of muscle-infiltrating/resident cells isolated 2 weeks and 6 weeks after immunization suggested a modest decrease in inflammatory cell burden (based on reduced proportion of CD45^+^ to CD45^–^ cells; data not shown), with contraction of the T/NK, macrophage/DC, and B cell clusters over that time period (Figure 6B and Supplemental Figure 11A). Further subclustering of T/NK cells yielded 1,380 cells, including a subset of CD4^+^ TRMs that were annotated based on the same criteria as in Figure 3. Notably, the general proportion of CD4^+^ TRMs within the T/NK cluster was retained (Figure 6C and Supplemental Figure 11B). Furthermore, the overall gene expression profile of this subcluster demonstrated persistence of the CD4^+^ TRM phenotype from 2 weeks to 6 weeks after immunization (Figure 6D and Supplemental Table 1).

Consistent with these findings, flow cytometry also demonstrated a stable phenotype of cell surface marker expression characteristic of CD4^+^ TRMs over this time period (Supplemental Figure 12). Limited variations from the 2-week time point were observed among other cell types in the T/NK cluster, with slightly decreased naive cells (CD69^–^CCR7^+^) and a concomitant increase in CD69^+^CCR7^+^ inflammatory T cells likely marking TEMs/TCMs (Supplemental Figure 13). Similarly, the number of FoxP3^+^ cells was stable over the 2- to 6-week period. Finally, the ability to express IFN-γ at the protein level upon in vitro activation was also found to be stable in CD69^+^CCR7^–^CXCR6^+^CD4^+^ TRMs (Supplemental Figure 14). Though the overall phenotypic differences among subsets were subtle, they generally mirrored changes in the relative proportions of naive cells and TEMs/TCMs by sequencing analysis (Figure 6C). Most importantly, these results demonstrated CD4^+^ TRM stability over time at both the RNA and protein expression levels.

**Figure 7:**
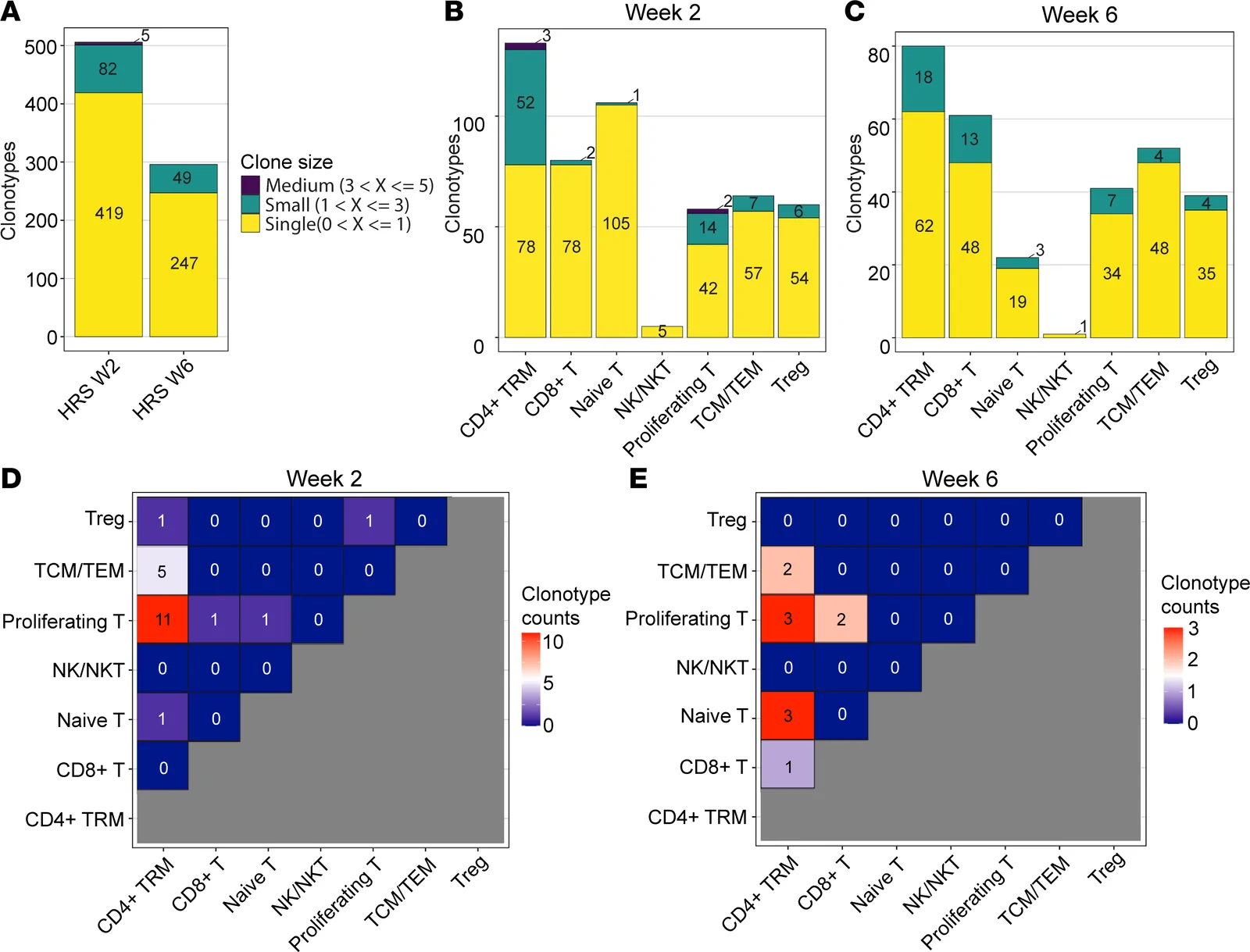
Expanded T cell clonotypes 2 weeks and 6 weeks after immunization. (**A**) Counts of T cell clonotypes (segregated by clonotype size) at 2 versus 6 weeks after immunization. (**B** and **C**) Counts of T cell clonotypes residing in 7 subsets of T/NK cells 2 weeks (**B**) and 6 weeks (**C**) after immunization. (**D** and **E**) Shared clonotypes between designated subclusters of T/NK cells 2 weeks (**D**) and 6 weeks (**E**) after immunization.

Given the persistence of (presumed) antigen-experienced CD4^+^ TRMs in muscle tissue over time (demonstrated by both scRNA-seq and flow cytometry), clonal expansion was examined at the 6-week post-immunization time point through combined TCR and RNA sequencing. Clonal expansion decreased from 2 weeks to 6 weeks (Figure 7A), indicating an overall reduction in inflammation within the muscle. Nevertheless, at both the 2-week and 6-week time points, most of the expanded clonotypes resided within the CD4^+^ TRM population (Figure 7, B and C). At 2 weeks after HRS immunization, 11 clonotypes were shared between proliferating T cells and CD4^+^ TRMs, while this number decreased to only 3 at the 6-week time point (Figure 7, D and E). This relative contraction suggests that CD4^+^ TRMs proliferated and differentiated significantly less in the absence of additional exogenous antigen stimulation. Furthermore, there were no shared clonotypes within the CD4^+^ TRM cell compartment when mice harvested 2 versus 6 weeks after immunization were compared, which might suggest dynamic attrition and recruitment in the context of declining numbers of TRMs over an extended period. Unfortunately, because individual mice could not be assessed at both 2 weeks and 6 weeks after immunization via sequential muscle biopsies (for technical reasons), it was not possible to determine whether CD4^+^ TRMs expressing a particular TCR would have been maintained over time.

## Discussion

Our findings provide a comprehensive characterization of CD4^+^ TRMs as key players in the immune response associated with murine HRS-induced myositis. By integrating scRNA-seq and TCR sequencing (TCR-seq), we demonstrated that these cells possess a canonical TRM transcriptional profile and undergo robust in situ clonal expansion. Coupled with their intensive signaling crosstalk with innate immune cells via IFN-γ and TNF signaling pathways, the persistence of clonally expanded CD4^+^ TRMs at later stages post-immunization suggests that they are not merely transient infiltrates but a stable, pathogenic reservoir. Given the striking overlap between these observations in our murine model and the transcriptional profile of human myositis specimens, these results underscore the potential role of IFN-γ–producing CD4^+^ TRMs as key mediators of muscle inflammation in the anti-synthetase subset of human IIM.

While the presence of TRMs has been noted across a limited number of IIM subtypes assessed by scRNA-seq (34), our study provides a targeted analysis focused on the anti-synthetase syndrome. Bulk RNA sequencing data from this subset of IIM patient biopsies suggested the presence of TRMs based on elevated expression of recruitment and retention markers (including *CD69*, *CXCR6*, and *CXCR3*) (Figure 1, A and C) as well as markers of robust type II interferon signaling that was most pronounced in the Jo-1 anti-synthetase subset (Figure 1D and Supplemental Figure 1). Expression of IFN-γ by TRMs has been reported in Sjögren’s syndrome (58), spondyloarthritis (59), and myositis (34), all diseases in which this cytokine has a known pathogenic function (60, 61). In our model system, the critical role of IFN-γ was substantiated by both scRNA-seq (Figure 3, A–D) and flow cytometry (Figure 3E). Because TRM signatures are often heterogeneous and tissue specific, this multimodal approach with single-cell resolution is critically important for distinguishing TRMs from other infiltrating lymphocyte populations (40).

The significance of IFN-γ in CD4^+^ TRMs was further indicated by cell-cell communication analysis in our study. Ligand-receptor computational analysis suggested that IFN-γ signals are transduced exclusively from CD4^+^ TRMs to macrophages or DCs. This finding reflects previous research by our group, in which IFN-γ–mediated signals play a prominent role in the crosstalk between T cells and macrophages (5). Given that IFN-γ is critical in driving macrophages toward a proinflammatory phenotype and increasing antigen presentation via STAT1 activation (62), our current data strongly suggest that CD4^+^ TRMs can also regulate components of the innate immune response (Figure 4A). At the same time, our previously published NicheNet analyses indicating that T cell–derived IFN-γ mediates crosstalk with fibroblasts (5) suggest that TRMs also play a role in driving fibroblasts toward a proinflammatory phenotype.

Consistent with the central role of IFN-γ signaling in this disease process, we have also observed that IFN-γ receptor–deficient (*Ifngr^–/–^*) mice failed to develop significant muscle inflammation following immunization with recombinant HRS (data not shown). In turn, the dependence of our model on IFN-γ signaling underscores the importance of TRMs, as these cells are a major source of this cytokine (Figure 3D and Figure 4G) that can impact both innate and adaptive immune responses.

TCR sequencing results revealed clonally expanded CD4^+^ TRM populations, consistent with an antigen-driven process mediated by HRS. The identification of shared TCR (TCRα/TCRβ) CDR3 sequences in different experimental groups suggests that these cells may recognize a common set of autoantigenic epitopes (Figure 5C). In spite of the limited number of shared clonotypes with identical TCR sequences, the number of shared clonotype clusters provides compelling evidence of convergent antigen specificity (Figure 5C and Supplemental Table 6). Additionally, the existence of shared clonotypes between CD4^+^ TRMs and proliferating T cell subsets suggests that the TRM pool is maintained or expanded through local proliferation, a conclusion further supported by overlapping expression of TRM marker genes (Figure 3D and Supplemental Figure 5, A and B). This active turnover further demonstrates that CD4^+^ TRMs are dynamic components of the local inflammatory environment rather than stagnant memory cells.

Once established, TRMs are generally thought to persist in tissues for the life of the host, but importantly, they have the capacity to become activated and expand rapidly — either in the context of an inflammatory milieu, such as in the presence of IL-15, or upon encountering specific antigens (63). The maintenance of CD4^+^ TRMs involves active self-renewal and continuous replacement within resident tissues (64). In the absence of cognate antigen sensing for a relatively extended period of time, CD4^+^ TRMs likely still decline (63), but again, some cells are thought to persist indefinitely. In our murine myositis model, the CD4^+^ TRM phenotype remains remarkably consistent over time and is characterized by the stable expression of *Cd69* and *Cxcr6* and the absence of *S1pr1* and *Sell* (Figure 6D). Although scRNA-seq analysis suggested a slight decrease in CD4^+^ TRM counts from 2 weeks to 6 weeks after immunization, flow cytometry demonstrated that the CD4^+^CD69^+^CCR7^–^CXCR6^+^ TRM population remained relatively stable from short- to long-term observation (Supplemental Figure 12). These results collectively indicate a minor contraction phase followed by a durable state of tissue residency. TRMs actively differentiate into TEMs and TCMs during inflammation, but exit to the recirculation pool as ex-TRMs (65, 66). Following the resolution of infection and in the absence of chronic antigen exposure, the initial proliferative burst of TRM cells gradually subsides, yielding a persistent population maintained by the muscle microenvironment, even without continuous antigen exposure and TCR signaling (67).

Despite the insights provided by this study, several limitations warrant consideration. First, the small sample size, which is a common constraint in muscle biopsy research, restricted the depth of our TCR-seq analysis. Although our data strongly suggest convergent antigen specificity, future studies using larger cohorts and functional validation techniques, such as MHC multimer profiling, are necessary to identify cognate antigens.

Notably, our observations are heavily skewed toward CD4^+^ TRMs, running counter to the current literature that is focused primarily on CD8^+^ TRMs. While this bias could reflect the overall predominance of CD4^+^ T cells in our model, it is important to note that scRNA-seq of human muscle biopsy specimens does reveal the presence of CD4^+^ TRMs, particularly in the Jo-1^+^ myositis subset (Supplemental Figure 6). Importantly, the transcriptional profile of CD4^+^ TRMs in HRS-induced myositis mirrors that described for CD8^+^ TRMs in other diseases — underscoring the fluidity of the TRM phenotype (which can vary by tissue type as well as disease state) (25, 68) and suggesting that transcriptional programming, rather than CD4 versus CD8 expression, is the key determinant of TRM function. As further evidence of this fluidity, a substantial percentage of CD8^+^ T cells isolated from muscle tissue in our model of HRS-induced myositis do have a CD69/CD44/CXCR6/CD103 cell surface phenotype that could be construed as “TRM-like.” Based on these observations, further investigation into specific functional subsets of TRMs and dissection of TCM/TEM profiles using higher resolution analyses could provide a more nuanced understanding of the local immune microenvironment in myositis. Crucially, to fully elucidate the role of CD4^+^ TRMs in myositis pathogenesis, integration of metabolomic profiling and targeted functional assays will be vital for future investigations into how these cells adapt to the inflammatory muscle milieu.

Notwithstanding these potential limitations, this study provides overall validation of our model of HRS-induced myositis as a robust proxy for human disease. In addition to histopathological hallmarks of the anti-synthetase syndrome (34, 69), our model mirrors the transcriptomic landscape of human IIM. Specifically, bulk RNA sequencing revealed similar expression of TRM signature genes in human anti–Jo-1 myositis and murine muscle tissue (Figure 1A and Supplemental Figure 2A), a key finding that was further validated at single-cell resolution in our mouse model of HRS-induced myositis (Figure 3, C and D). Consequently, although our model does not replicate all of the functional characteristics of human disease (such as persistent muscle weakness), it does provide a reliable platform for longitudinal investigation of TRM dynamics and screening of novel therapeutics targeting the IFN-γ axis.

In conclusion, our study leverages multimodal single-cell sequencing to identify a clonally expanded and transcriptionally distinct CD4^+^ TRM population that acts as a key source of IFN-γ in murine myositis. Our findings demonstrate that TRM-derived signaling is not merely an auxiliary feature, but a pivotal driver of the inflammatory cascade of tissue-resident memory in chronic autoimmunity. These results offer critical insights into the immune landscape of IIM and underscore the importance of TRM dissection in the pursuit of targeted therapeutic interventions.

## Methods

### Sex as a biological variable.

Our study exclusively examined female mice because the disease model has been established in female mice and because human disease is female-predominant. Human muscle tissue specimens were obtained from both males and females.

### Bulk RNA sequencing analysis of human IIM muscle biopsies.

Bulk RNA sequencing was performed as previously described (70–72) on 662 frozen human muscle biopsy specimens from a National Institute of Arthritis and Musculoskeletal and Skin Diseases, National Institutes of Health, biorepository. These specimens consisted of 37 anti–HRS/Jo-1 samples, 37 healthy controls, and 588 additional samples encompassing dermatomyositis (DM), immune-mediated necrotizing myopathy (IMNM), and inclusion body myositis (IBM). Libraries were prepared with either the NeoPrep system according to the TruSeqM Stranded mRNA Library Prep protocol (Illumina), or the NEBNext Poly(A) mRNA Magnetic Isolation Module and Ultra II Directional RNA Library Prep Kit for Illumina (E7490 and E7760, New England Biolabs). Reads were demultiplexed using bcl2fastq 2.20.0 (https://support.illumina.com/downloads/bcl2fastq-conversion-software-v2-20.html) and preprocessed using fastp 0.21.0 (https://github.com/OpenGene/fastp). The abundance of each gene was determined using Salmon 1.5.2, and quality control output was summarized using multiqc 1.11. Counts were normalized using the trimmed means of M values (TMMs) from edgeR 3.34.1 for graphical analysis. Differential expression was performed using the limma-voom method. Gene set enrichment analysis (GSEA) was performed using the WebGestalt tool to identify enrichment of genes in specific pathways (73).

### Flex scRNA-seq analysis of human IIM muscle biopsies.

Single-cell transcriptome libraries were generated from formalin-fixed, paraffin-embedded human muscle biopsy specimens (*n* = 16) using the 10x Genomics Chromium Single Cell Flex assay, a probe-based approach designed to profile fixed cells. Libraries prepared with this approach were sequenced at a depth of approximately 10,000 mean reads per cell, in line with 10x Genomics’ recommendations for Flex gene expression datasets. After sequencing of Flex samples, FASTQ files were processed with the 10x Cell Ranger multiplex Flex (1 probe barcode per sample) workflow, performing alignment, filtering, and unique molecular identifier counting through the “multi” function and outputting per-sample gene-barcode count matrices. The reference human genome GRCh38 was used for mapping along with the associated 10x Genomics probe set, Chromium Human Transcriptome v1. Further analyses were carried out using the Seurat v5 R package.

### Murine strains and experimental myositis induction.

Mice used in this study were all derived from C57BL/6J (catalog 000664, The Jackson Laboratory) and were used at 8–10 weeks of age. In order to induce experimental myositis, 100 μL of affinity-purified, filter-sterilized recombinant HRS (3–5 mg/mL) was administered to the hamstrings bilaterally (50 μL/side). Two groups of mice were sacrificed at early (2 weeks) or late (6 weeks) time points post-immunization. Muscle tissue, serum, draining lymph nodes, and spleens were collected and archived appropriately for future analysis. All animal husbandry and experimental procedures were performed according to Association for Assessment and Accreditation of Laboratory Animal Care guidelines, and animal protocols were approved by the University of Pittsburgh Institutional Animal Care and Use Committee.

### Single-cell preparation and RNA sequencing of murine muscle.

Muscle-infiltrating cells were isolated from pooled groups of 3–5 mice immunized with recombinant HRS versus PBS (immunization described above) through conventional tissue dissociation techniques, and RNA sequencing was performed following the methods previously published by our group (5). In brief, single-cell suspensions were generated from hind limb mouse muscle tissue using the Miltenyi Biotec Skeletal Muscle Dissociation Kit and a gentleMACS Octo Dissociator (Miltenyi Biotec), filtered, and resuspended in PBS. Suspensions were then mixed with reverse transcription reagents and loaded into a Chromium Controller instrument (10x Genomics) for cDNA preparation. cDNA libraries were sequenced on the NovaSeq 6000 platform and aligned to 10x Genomics’ mouse references using Cell Ranger 6.1.2. Two aliquots of the amplified cDNA were combined with 10x Genomics mouse T Cell Mix and B Cell Mix to profile the TCRs and BCRs of muscle-infiltrating lymphocytes. Library preparation and sequencing were performed in separate batches for independent experiments (in which paired samples from HRS-immunized and control mice were sequenced together).

### Spectral flow cytometry.

Immune profiling of muscle-infiltrating cells was assessed using spectral flow cytometry. Staining for cell surface and intracellular markers was performed by conventional methods. Briefly, single-cell suspensions were prepared from inflamed muscle tissue and were stained (10^6^ cells per reaction) in a total volume of 100 μL of flow cytometry buffer (FACS buffer; PBS plus 2% fetal bovine serum). The cells were incubated with fixable viability dye (FVD) eFluor 506 (catalog 65-0866, Invitrogen/eBioscience) for 30 minutes on ice followed by Fc Block (catalog 50-112-2773, Invitrogen) for an additional 10 minutes. Cell surface staining was then performed on ice for 30 minutes by the addition of a master mix made up of optimally diluted monoclonal antibodies (all from BioLegend): anti-CD45–PE-Cy5 (catalog 103110), anti-CD3–Alexa Fluor 488 (catalog 100210), anti-CD4–BV711 (catalog 100557), anti-CD8–APC–Fire 810 (catalog 104007), anti-CD69–PE (catalog 164204), anti-CD103–BUV496 (catalog 741083), anti-CD44–BV421 (catalog 103040), anti-CD62L–APC (catalog 104412), anti-CCR7–BUV395 (catalog 740325), and anti-CXCR6–PE-Cy7 (catalog 151118). After sequential washing, the cells were prepared for intracellular staining using intracellular staining kit reagents (Invitrogen/eBioscience). The cells were fixed overnight at 4°C with fixation buffer and subsequently washed 3 times with FACS buffer and once with permeabilization buffer. Master mix containing anti-FoxP3–Alexa Fluor 700 (catalog 126421) and/or anti–IFN-γ–BV785 (catalog 505838) (both from BioLegend) in permeabilization buffer was then used to resuspend the cells, followed by a 30-minute incubation on ice. Finally, 2 washes with permeabilization buffer and 1 wash with FACS buffer were performed. Cells were resuspended in FACS buffer and were stored at 4°C in the dark until data acquisition (typically within 24 hours or less). Single-fluorochrome control samples for spectral unmixing were prepared using each of the aforementioned monoclonal antibodies and UltraComp eBeads compensation beads (Invitrogen). Muscle-infiltrating cells were used for unstained and FVD controls, the latter of which consisted of a mixture of untreated and heat-killed (2 minutes at 65°C) cells. In experiments for assessment of IFN-γ cytokine expression, cells were stimulated in vitro using a T cell activation/expansion kit (Miltenyi Biotec) for 72 hours at 37°C before staining, with phorbol myristate acetate (PMA)/ionomycin (MilliporeSigma), brefeldin A (Invitrogen), and monensin (Invitrogen) added during the final 2.5 hours of culture.

Data acquisition and spectral unmixing were performed using an Aurora 5-laser spectral flow cytometer (Cytek) running SpectroFlo software, housed within the University of Pittsburgh Department of Immunology United Flow Cytometry Core. Analysis was carried out using FlowJo software (Tree Star).

### scRNA-seq data processing and quality control.

The scRNA-seq data from week 2 post-immunization muscle biopsies were combined with published single-cell datasets (Gene Expression Omnibus GSE229059) and analyzed together. In total, 6 wild-type groups (HRS = 3, PBS = 3) and 2 *MyD88*-flox/flox groups (HRS = 1, PBS = 1) were included (each group contained pooled muscle-infiltrating cells from 3–5 mice, except where indicated). scRNA-seq data were analyzed in R using the Seurat package (version 5.3.0).

To remove doublets and low-quality data, cells with more than 4,000 or fewer than 500 detectable genes were filtered out. Cells with a high percentage of mitochondria genes were similarly eliminated from further analysis. The raw read counts were normalized using the NormalizeData function. Batch effects were removed with canonical correlation analysis using the IntegrateLayers function. To avoid unclear clustering caused by proliferating cells, cell cycle effects were scored (cc.genes.updated.2019) and removed during principal component analysis.

### Cluster analysis and gene expression profiling.

Based on the global transcriptional profile, we used FindNeighbors and FindClusters functions to identify cell clusters. Cell clusters were defined based on differentially expressed genes exhibiting log_2_ fold-changes ≥ 1, recognized by the FindMarkers function using a Wilcoxon’s rank-sum test. Because NKT cells were admixed with T and NK cells, the latter two populations could not be separated into distinct clusters and were therefore annotated as “T/NK” before subclustering analysis. GO and KEGG analyses were performed on the lists of differentially expressed genes obtained from the FindMarkers function using the clusterProfiler package (version 4.14.6).

### Trajectory analysis.

The trajectory analysis was conducted using Monocle 3 (cole-trapnell-lab.github.io/monocle3, version 1.4.26). All T/NK cell clusters were selected to construct the trajectory. The naive T cells were set as the root state of the trajectory in the order in which cells function.

### Cell-cell communication analysis.

Based on ligand and receptor gene expression profile, input and output signaling among the different cell types and cell clusters was assessed by the CellChat (74) package. The netAnalysis_signalingRole_heatmap function was used to visualize all detectable signaling pathways among cell clusters. netVisual_heatmap and netVisual_circle were used to evaluate signaling strength of cell-to-cell communication and to visualize signaling networks within specific subsets of cells. With regard to statistical analysis, interaction strength represents communication probabilities derived from a mass action model incorporating ligand-receptor expression. Incoming/outgoing signaling patterns describe the coordination between cell types and signaling pathways using pattern recognition approaches.

### TCR sequencing analysis.

For each sample, an output file was generated by Cell Ranger named “filtered_contig_annotations.csv,” which contains CDR3 (TCRα and TCRβ) nucleotide sequences. The scRepertoire package (version 2.5.2) (75) was used for further analysis. TCR-seq data from different samples were combined into a list object with the combineTCR function, and the TCR contig list was then integrated with the Seurat object of the scRNA-seq data using the combineExpression function. The count of the same clones was defined as cloneSize. The cloneSize was manually sorted into single = 1; 1 < small ≤ 3; 3 < medium ≤ 5; 5 < large ≤ 15 (no cloneSize reached higher than 15). The cloneSize and its frequency were added to scRNA-seq metadata. Clonotype networks within CD4^+^ TRMs were assessed and visualized using the clonalNetwork function, based on igraph (2.2.1). Multiple sequence alignment of TCR-β sequences was performed using Clustal Omega (European Molecular Biology Laboratory’s European Bioinformatics Institute [EMBL-EBI]; www.ebi.ac.uk/Tools/msa/clustalo) with default parameters. Results of alignment were visualized using WebLogo (https://weblogo.berkeley.edu).

### Statistics.

All statistics in this study were calculated in R. Human muscle bulk RNA sequencing data were analyzed using Partek Flow software (Illumina). Differential analysis *P* values were calculated using the limma-voom method and corrected with a false discovery rate (FDR) using the Benjamini-Hochberg method. For mouse scRNA-seq analysis, differential gene expression *P* values were calculated with a 2-sided Wilcoxon’s rank-sum test using the Seurat package in R and corrected with an FDR using the Bonferroni correction. Findings with an FDR less than 0.05 were considered significant.

### Study approval.

All animal experiments in this study were performed under protocols approved by the University of Pittsburgh Institutional Animal Care and Use Committee (25056776, 22030750). Human muscle tissue for scRNA-seq was obtained through University of Pittsburgh IRB protocol 19090054. Publicly available data corresponding to bulk RNA sequencing of human muscle tissue were initially obtained through an independent IRB protocol approved through the National Institutes of Health (NIH).

### Data availability.

Public databases of human myositis (GSE220915) were used to explore the significance of resident memory T cells in anti–Jo-1 myositis. Bulk RNA sequencing data corresponding to murine HRS-induced myositis were deposited in the Gene Expression Omnibus (GEO; NCBI) repository (GSE336063). scRNA-seq data of murine myositis are also available in the GEO database (GSE229059, GSE335841). Similarly, single-cell sequencing of human muscle tissue specimens was deposited in the GEO repository (GSE35819).

## Author contributions

DL, TBO, and DPA designed experiments. DL, DPR, IPF, MCD, TBO, and DPA performed experiments. DL, IPF, ALM, TBO, SLG, and DPA interpreted data. DL, TBO, and DPA drafted the manuscript. DL, DPR, IPF, ALM, SLG, TBO, and DPA edited the manuscript.

## Conflict of interest

The authors have declared that no conflict of interest exists.

## Funding support

This work is the result of NIH funding, in whole or in part, and is subject to the NIH Public Access Policy. Through acceptance of this federal funding, the NIH has been given a right to make the work publicly available in PubMed Central.

- NIH R01AR071369 (to DPA).

## Supplementary Material

Supplemental data

Supplemental table 1

Supplemental table 2

Supplemental table 3

Supplemental table 4

Supplemental table 5

Supplemental table 6

Supplemental table 7

## Version 1 07/23/2026

In-Press Preview

## Version 2 09/08/2026

Electronic publication
