# Supplementary material for: Single-cell RNA sequencing reveals clonally expanded CD4+ tissue-resident memory T cells in histidyl-tRNA synthetase–induced myositis
Source: JCI Insight. 2026 Jul 23;11(17):e208009. doi: 10.1172/jci.insight.208009 (PMC13564085; doi:10.1172/jci.insight.208009)
Supplement: Supplementary file 1 [file jciinsight-11-208009-s248.pdf]

Supplementary Materials

Single-Cell RNA sequencing reveals clonally-expanded CD4+ tissue-resident memory T cells in Histidyl-tRNA Synthetase-induced myositis

Authors: Decheng Li[1, 2], Daniel P. Reay[1]. Iago Pinal-Fernandez[3, 4], Maria Casal-Dominguez, [3,4] Andrew L. Mammen[3, 4, 5], Sarah L. Gaffen[1], Timothy B. Oriss[1], and Dana P. Ascherman[1, *]

This document includes:

Supplementary Figures 1-12

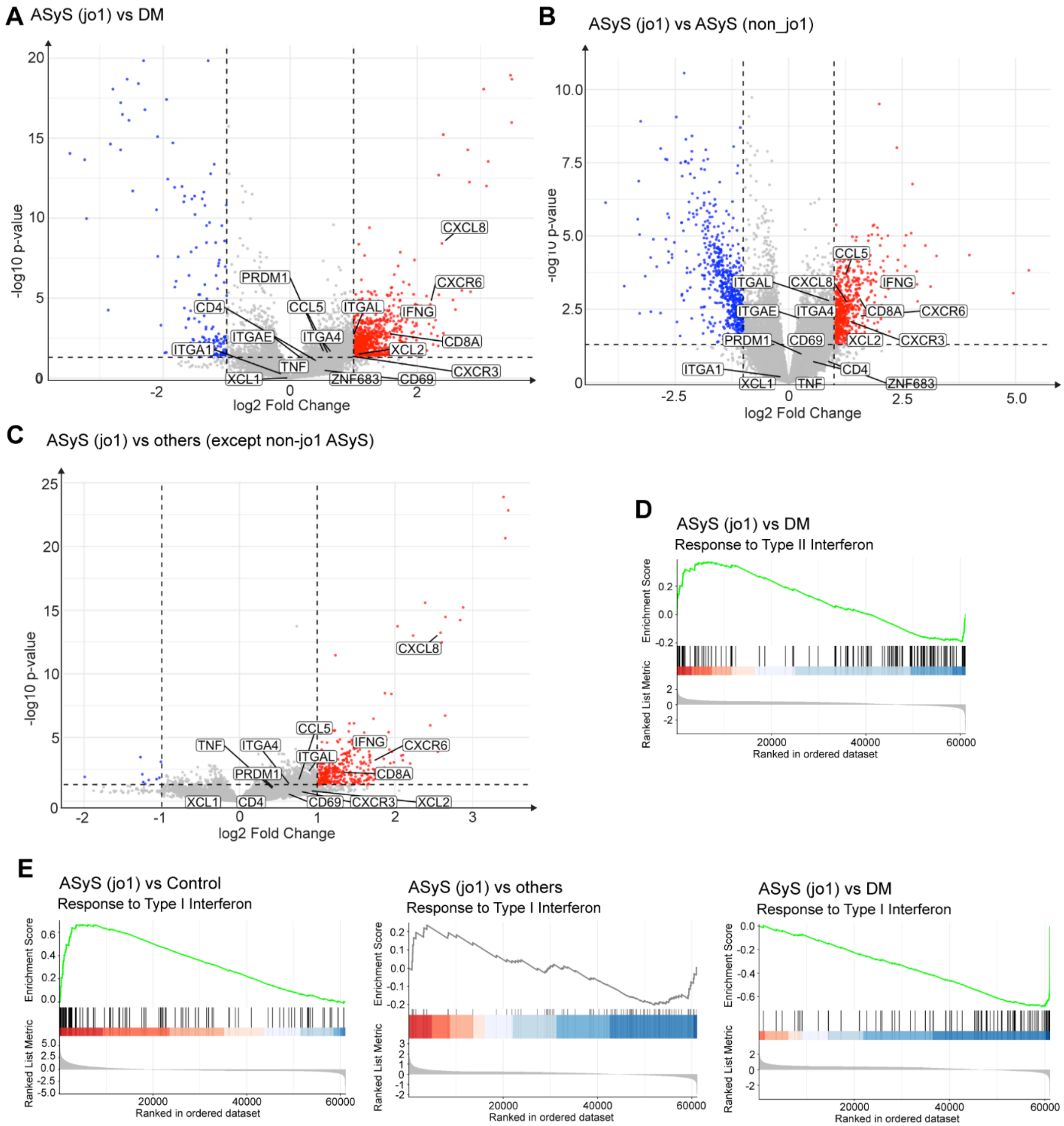

Supplementary Figure 1. Bulk RNA sequencing analysis of muscle biopsy from human myositis. Volcano plots display differentially expressed genes (DEGs) in anti-Jo-1 myositis patients (n = 37) compared to dermatomyositis (DM) (n = 105) (A), non-Jo-1 anti-synthetase syndrome (ASyS) (n = 28) (B), and all other myositis subtypes except non-Jo-1 ASyS (n = 560) (C). Genes with log2 FC > 1 and adjusted p < 0.05 were designated as upregulated; those with log2 FC < -1 and adjusted p < 0.05 were classified as downregulated. (D) Illustration of gene set enrichment (GSEA) for response to type II interferon biological process, identified from GO Enrichment, comparing anti-Jo-1 ASyS to DM. (E)

Illustration of gene set enrichment (GSEA) for response to type I interferon biological process, identified from GO Enrichment, comparing anti-Jo-1 ASyS to healthy controls, all other myositis, and DM.

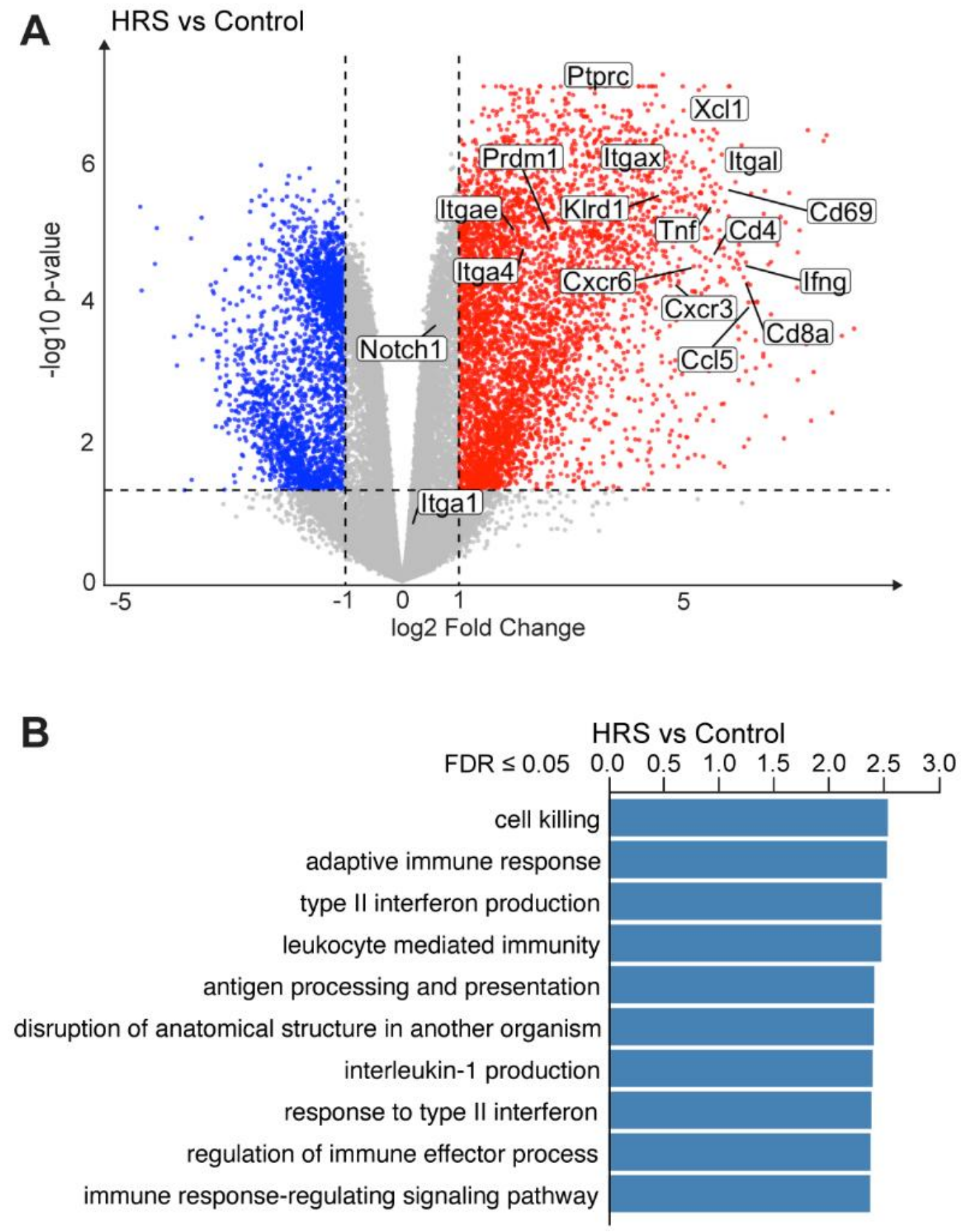

Supplementary Figure 2. Bulk RNA sequencing analysis of HRS-induced murine myositis. (A) Volcano plot displaying DEGs in HRS-induced myositis compared with control group. Genes with log2 FC > 1 and adjusted $p < 0.05$ were designated as upregulated; those with log2 FC < -1 and adjusted $p < 0.05$ were classified as downregulated. (B) The bar plot (right) demonstrates the top 10 pathways enriched from Gene Ontology (GO) analysis comparing HRS-induced myositis to controls, ordered by normalized enrichment scores. FDR < 0.05, minimum gene IDs in category = 20.

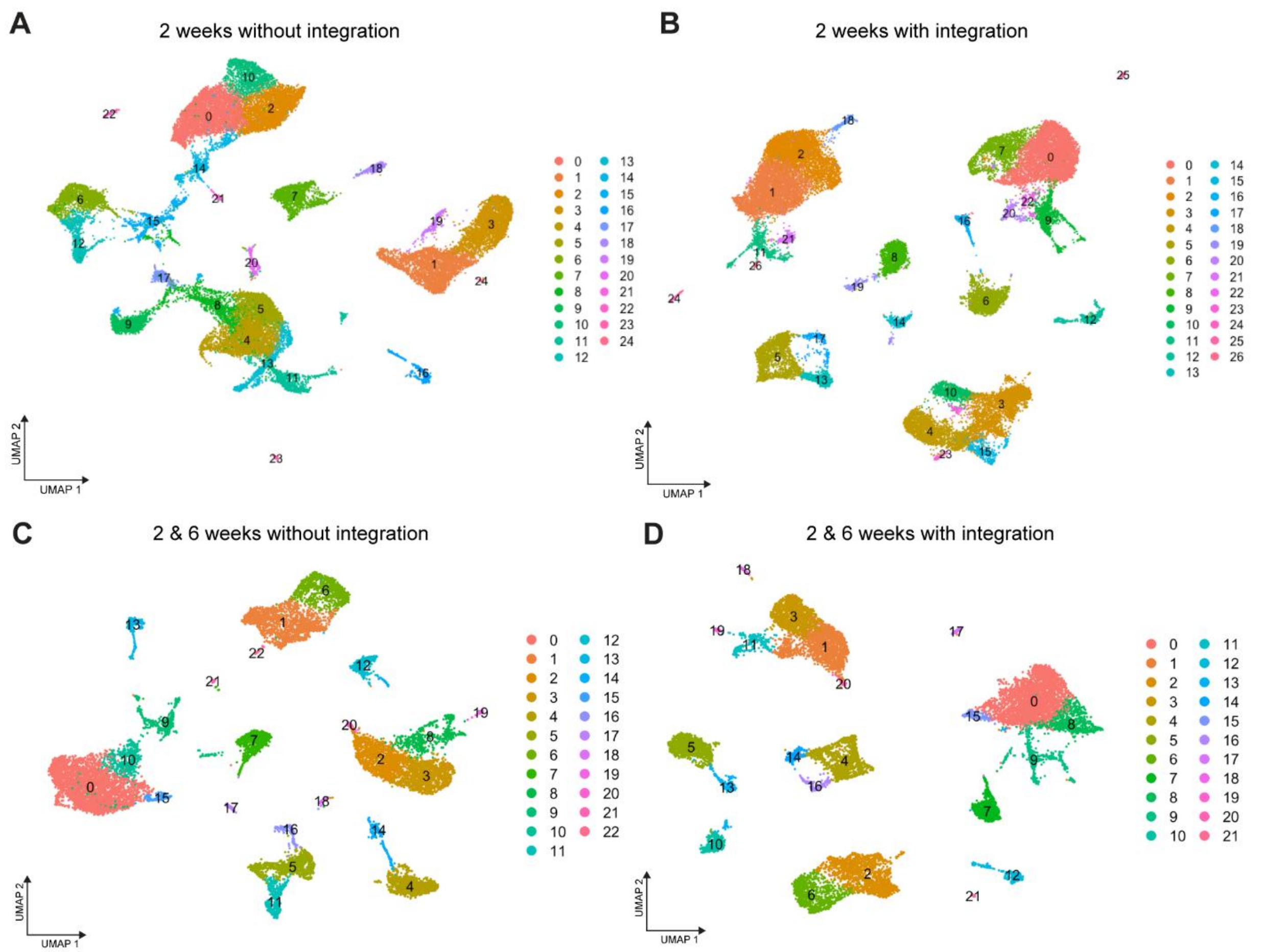

Supplementary Figure 3. Batch effect removal in scRNA-seq analysis. (A-B) scRNA-seq analysis for 2-week immunization before and after integration. All 2-week immunization samples are included (indicated in methods). (A) UMAP visualization without sample integration (A) , showing 24 clusters identified in muscle-infiltrating cells from mice with histidyl-tRNA synthetase (HRS)-induced myositis at 2 weeks post immunization. (B) UMAP visualization after sample integration and batch effect removal for 2-week immunization. Annotations of each cluster are provided in **Figure 2** and in the text of the manuscript. (C, D) scRNA-seq analysis for 2-week and 6-week immunization before and after integration. Only the 2-week experiments conducted concurrently with 6-week samples are included. (C) UMAP visualization without sample integration, showing 22 clusters identified in muscle-infiltrating cells from mice with HRS-induced myositis at both 2-week and 6-week time points. (D) UMAP visualization after sample integration and batch effect removal at 2-week and 6-week time points. Annotations of each cluster are provided in **Figure 6** and in the text of the manuscript.

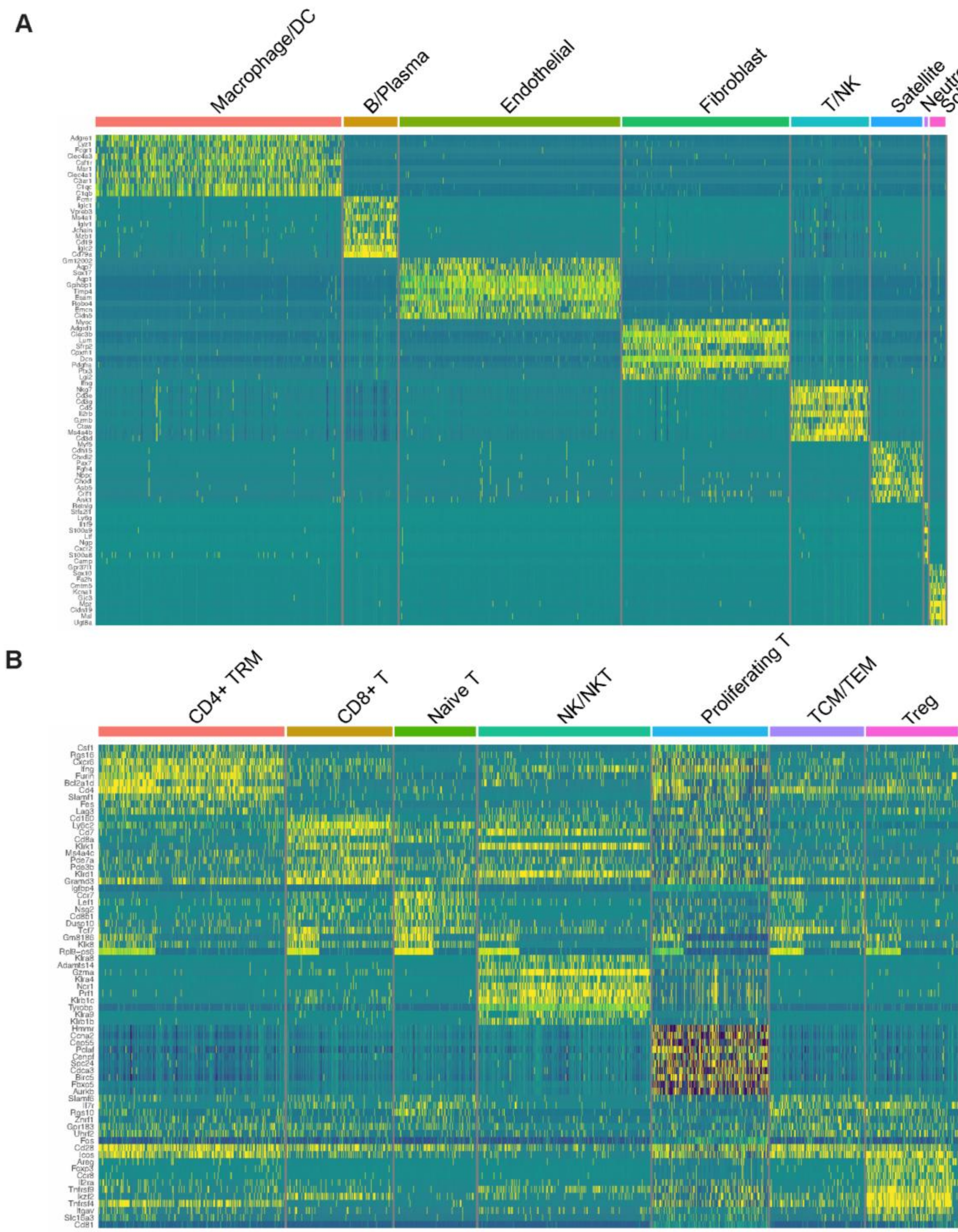

Supplementary Figure 4. Heatmap for cluster annotations in single-cell RNA sequencing. Heatmaps showing normalized expression of top 10 differentially expressed genes from all clusters (A) and from subclusters within T/NK cells (B).

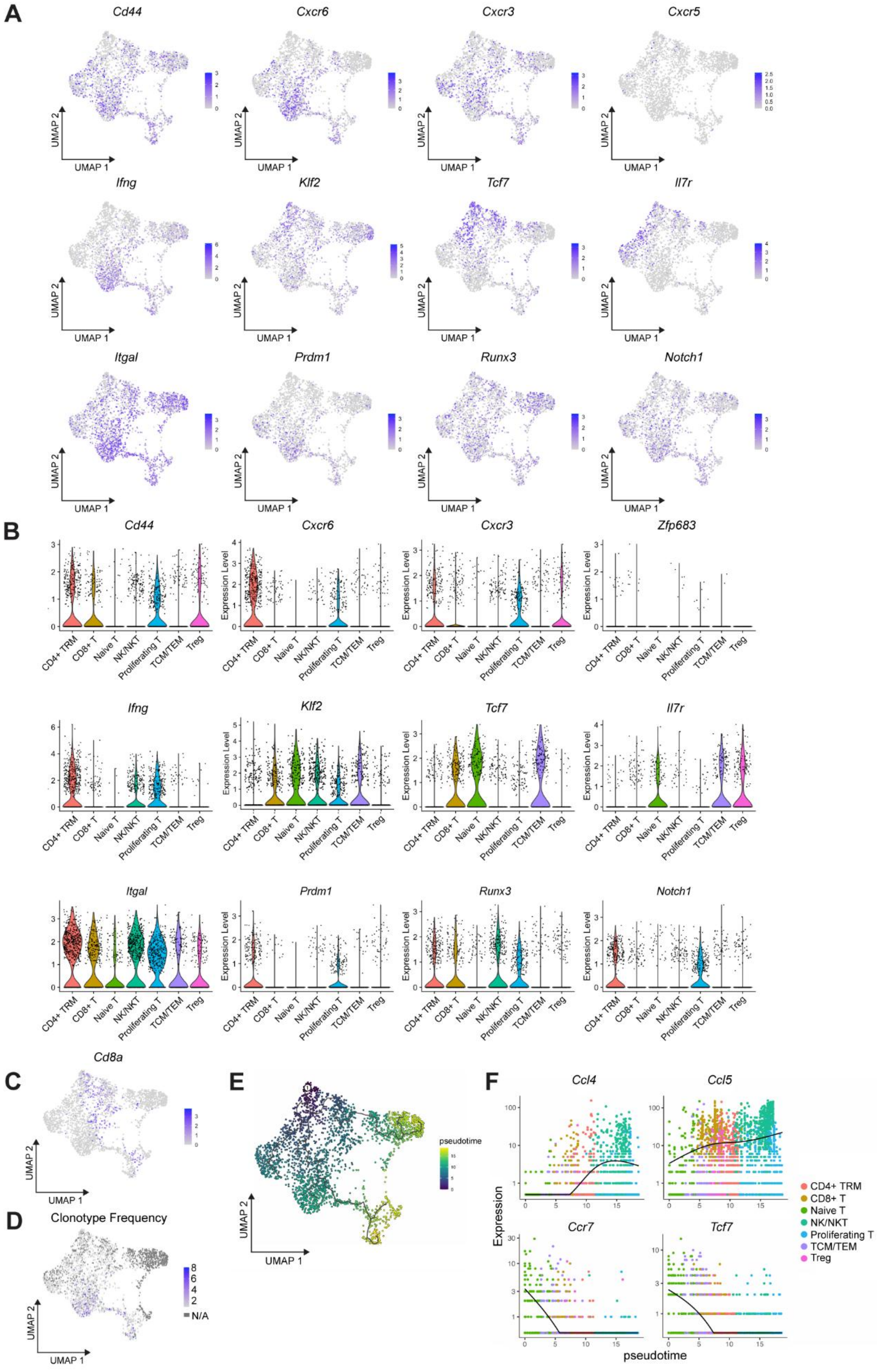
A
Cd44
Cxcr6
Cxcr3
Cxcr5
Ifng
Klf2
Tcf7
Il7r
Itgal
Prdm1
Runx3
Notch1
UMAP 1
UMAP 2
B
Cd44
Cxcr6
Cxcr3
Zfp683
Ifng
Klf2
Tcf7
Il7r
Itgal
Prdm1
Runx3
Notch1
Expression Level
CD4+ TRM
CD8+ T
Naive T
NK/NKT
Proliferating T
TCM/TEM
Treg
C
Cd8a
D
Clonotype Frequency
N/A
E
pseudotime
F
Ccl4
Ccl5
Ccr7
Tcf7
Expression
pseudotime
CD4+ TRM
CD8+ T
Naive T
NK/NKT
Proliferating T
TCM/TEM
Treg

Supplementary Figure 5. Marker gene expression and dynamics across cell trajectories in HRS-induced myositis. (A, B) Normalized expression of signature genes visualized in feature plots (A) and violin plots (B), with T cell subclusters corresponding to the UMAP projection shown in **Figure 3A**. (C) Normalized expression of *Cd8a* gene visualized in feature plot. (D) Frequency of clonotypes visualized in feature plots, scaled from low (grey) to high (purple). Cells with no clonotypes detected are indicated by dark dots. (E) Pseudotime value visualized in feature plot. The UMAP corresponds to visualization in Figure 3A. (F) Normalized expression of *Ccl4*, *Ccl5*, *Ccr7*, and *Tef7* genes visualized as a function of pseudotime.

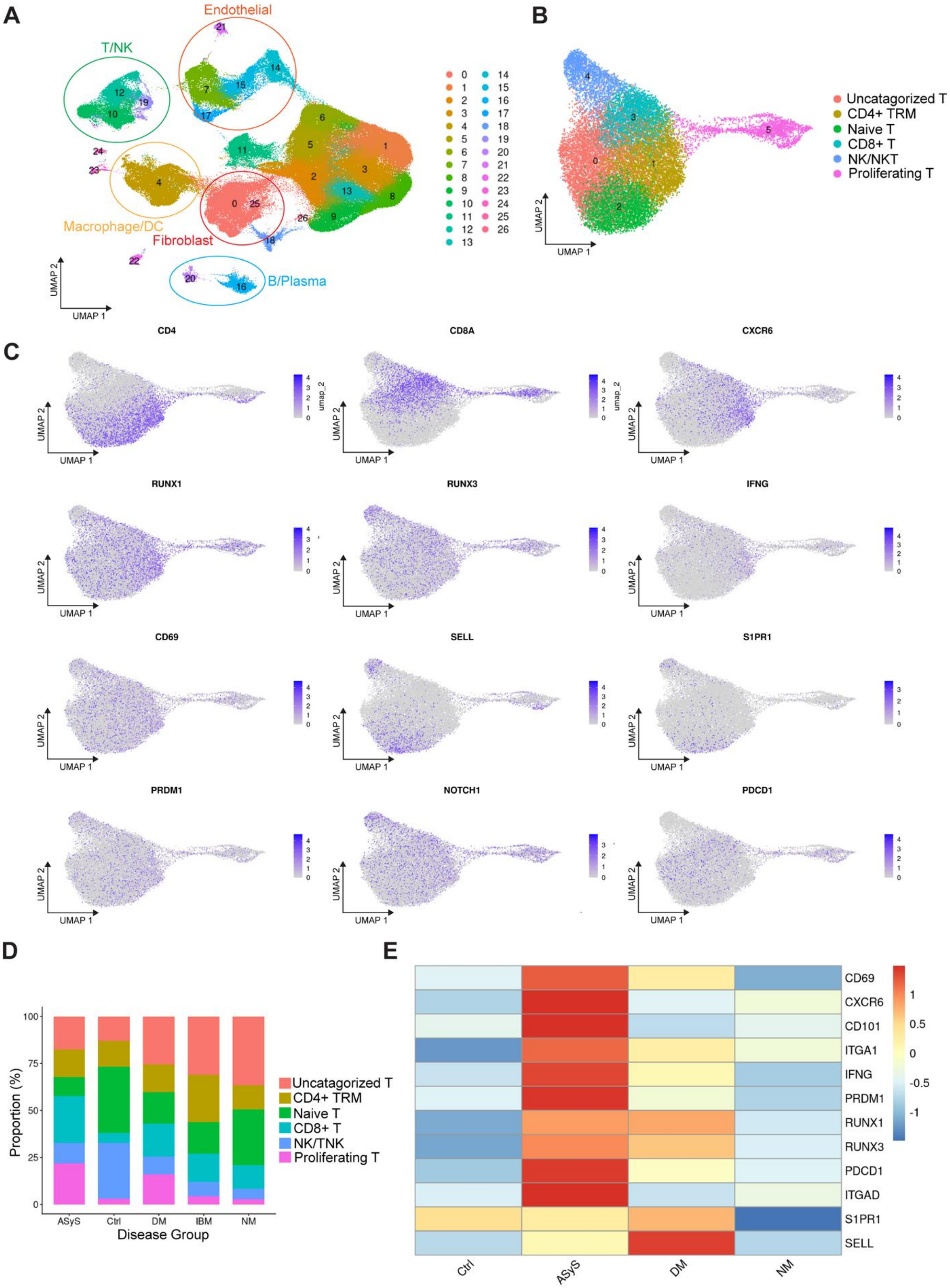
A
Endothelial
T/NK
Macrophage/DC
Fibroblast
B/Plasma
UMAP 1
UMAP 2
B
Uncatagorized T
CD4+ TRM
Naive T
CD8+ T
NK/NKT
Proliferating T
C
CD4
CD8A
CXCR6
RUNX1
RUNX3
IFNG
CD69
SELL
S1PR1
PRDM1
NOTCH1
PDCD1
D
Proportion (%)
Disease Group
ASyS
Ctrl
DM
IBM
NM
Uncatagorized T
CD4+ TRM
Naive T
CD8+ T
NK/TNK
Proliferating T
E
CD69
CXCR6
CD101
ITGA1
IFNG
PRDM1
RUNX1
RUNX3
PDCD1
ITGAD
S1PR1
SELL
Ctrl
ASyS
DM
NM

Supplementary Figure 6. Human scRNA-seq analysis of CD4+ TRM signature. (A) UMAP visualization of 26 clusters identified in infiltrating cells isolated from muscle tissue corresponding to n=3 Jo-1 ASyS, n=3 DM, n=3 IMNM, n=3 IBM, and n=4 non-myositis control specimens. Cluster annotations corresponding to related cell subsets are indicated by labeled circles. (B) UMAP visualization of 6 clusters identified from the T/NK cluster identified in (A). 0: uncategorized T cells; 1: CD4+ TRMs; 2: Naïve T cells; 3: CD8+ T cells; 4: NK/TNK cells; 5: Proliferating T cells. (C) Normalized expression of TRM signature genes visualized in feature plots. (D) Percentage of each T cell subcluster contained within muscle biopsy specimens representing indicated disease subsets. (E) Pseudobulk analysis of CD4+ TRM (subcluster 1, panel B) gene expression, comparing ASyS, DM, IMNM (NM), and healthy controls.

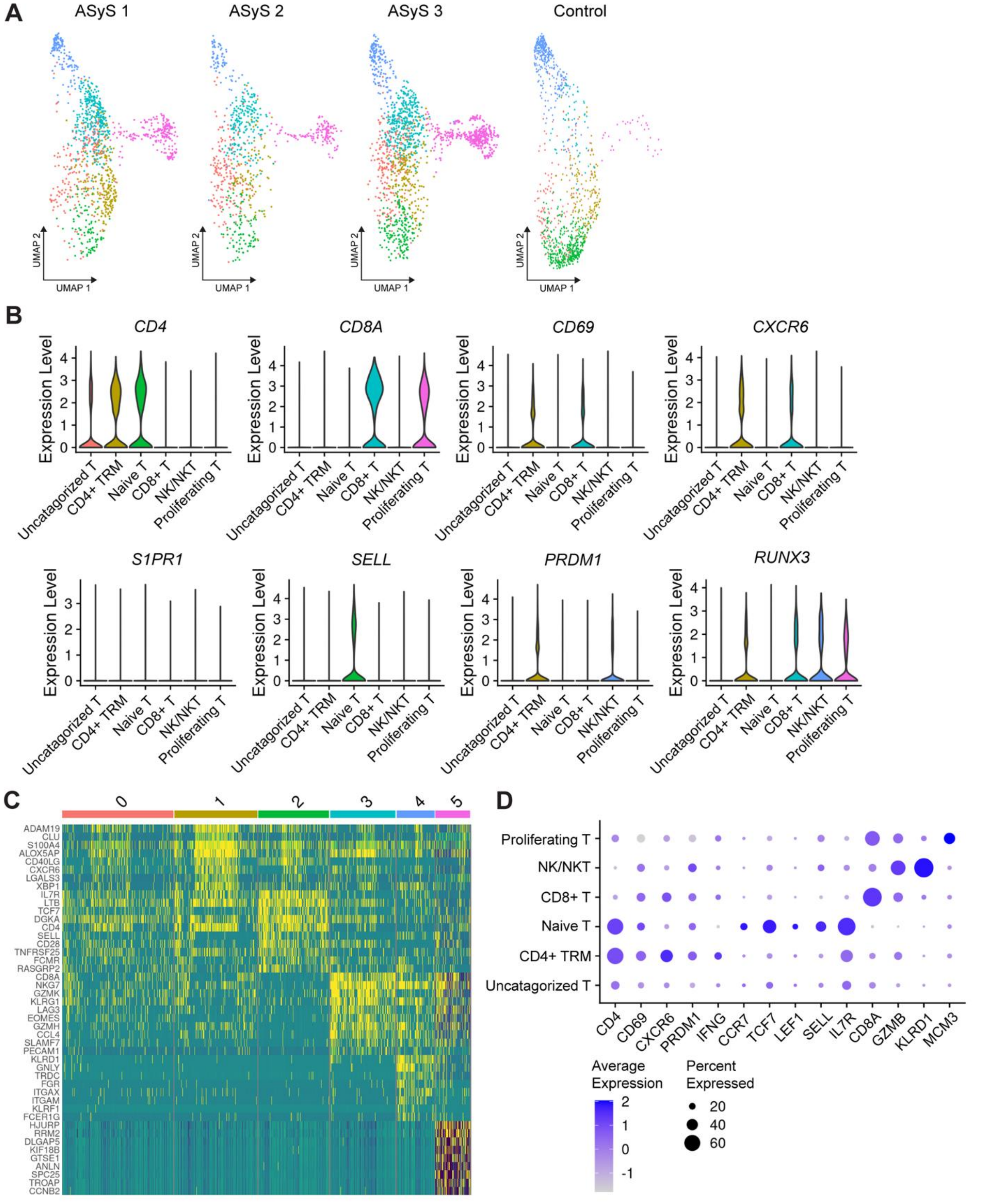

Supplementary Figure 7. CD4+ TRM annotation from human muscle scRNAseq, (A) UMAP visualization of the T/NK cluster identified from **Supplementary Figure 6A**, demonstrating 3 individual muscle samples corresponding to anti-synthetase syndrome (ASyS) as well as a composite control group (n=4 muscle specimens). (B) Expression of CD4+ TRM marker genes in

different T/NK subsets, visualized in violin plots. (C) Top 10 DEGs of T/NK subsets. 0: uncategorized T cells; 1: CD4+ TRMs; 2: Naïve T cells; 3: CD8+ T cells; 4: NK/TNK cells; 5: Proliferating T cells. (D) Dot plot demonstrating DEGs in T/NK subsets.

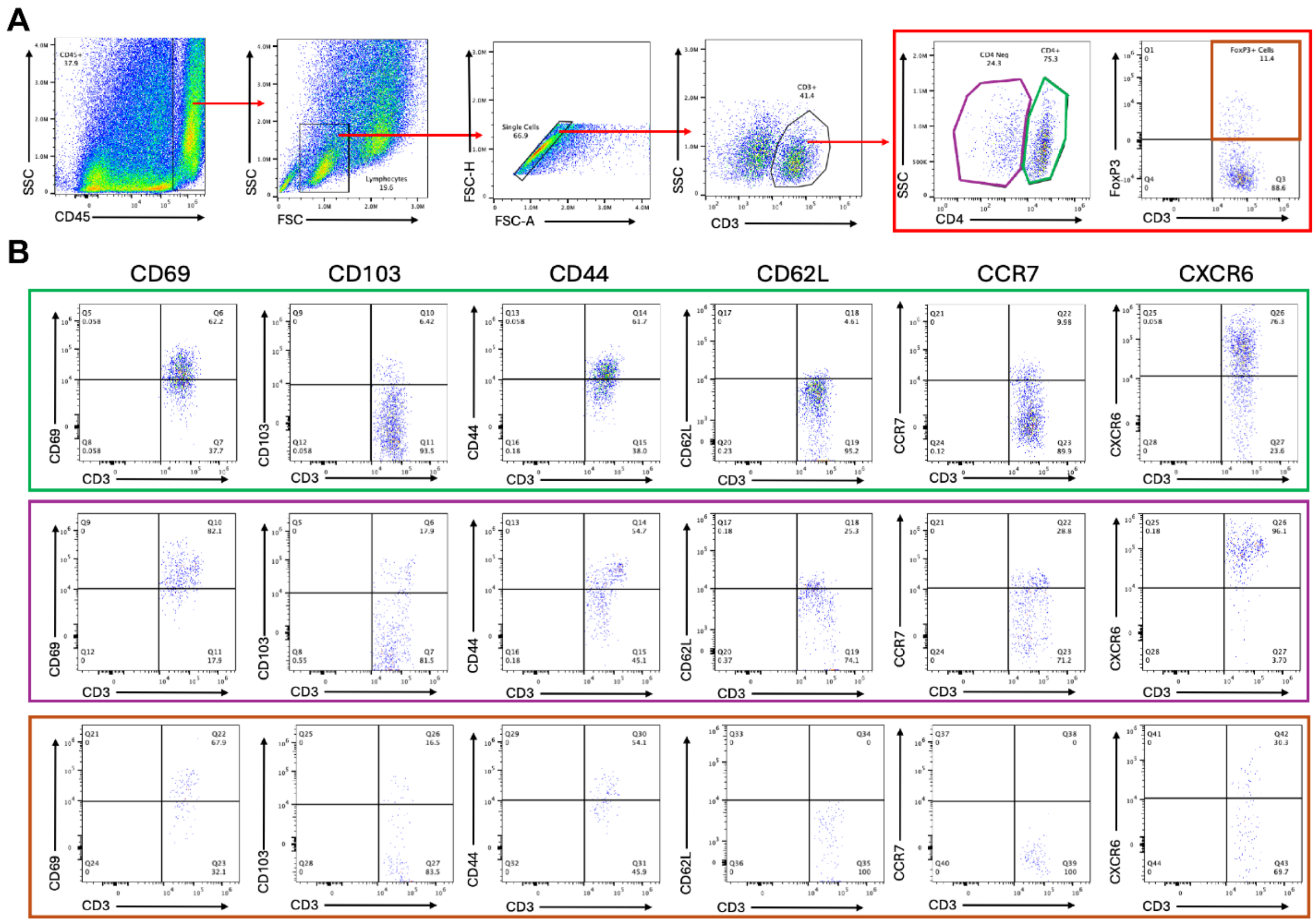

Supplementary Figure 8. T cell sub-populations and cell surface phenotype of muscle-infiltrating inflammatory cells. Spectral flow cytometry was used to assess T cell subpopulations two weeks after intramuscular injection with HRS. Viable muscle cells (fixable viability dye eFluor 506) were gated as shown in (A) in order to identify CD4+ (green gate), CD4- (verified to be CD8+; not shown; purple gate), and FoxP3+ (brown gate) cells. Each of the subpopulations were then phenotyped based on expression of the indicated cell surface markers.

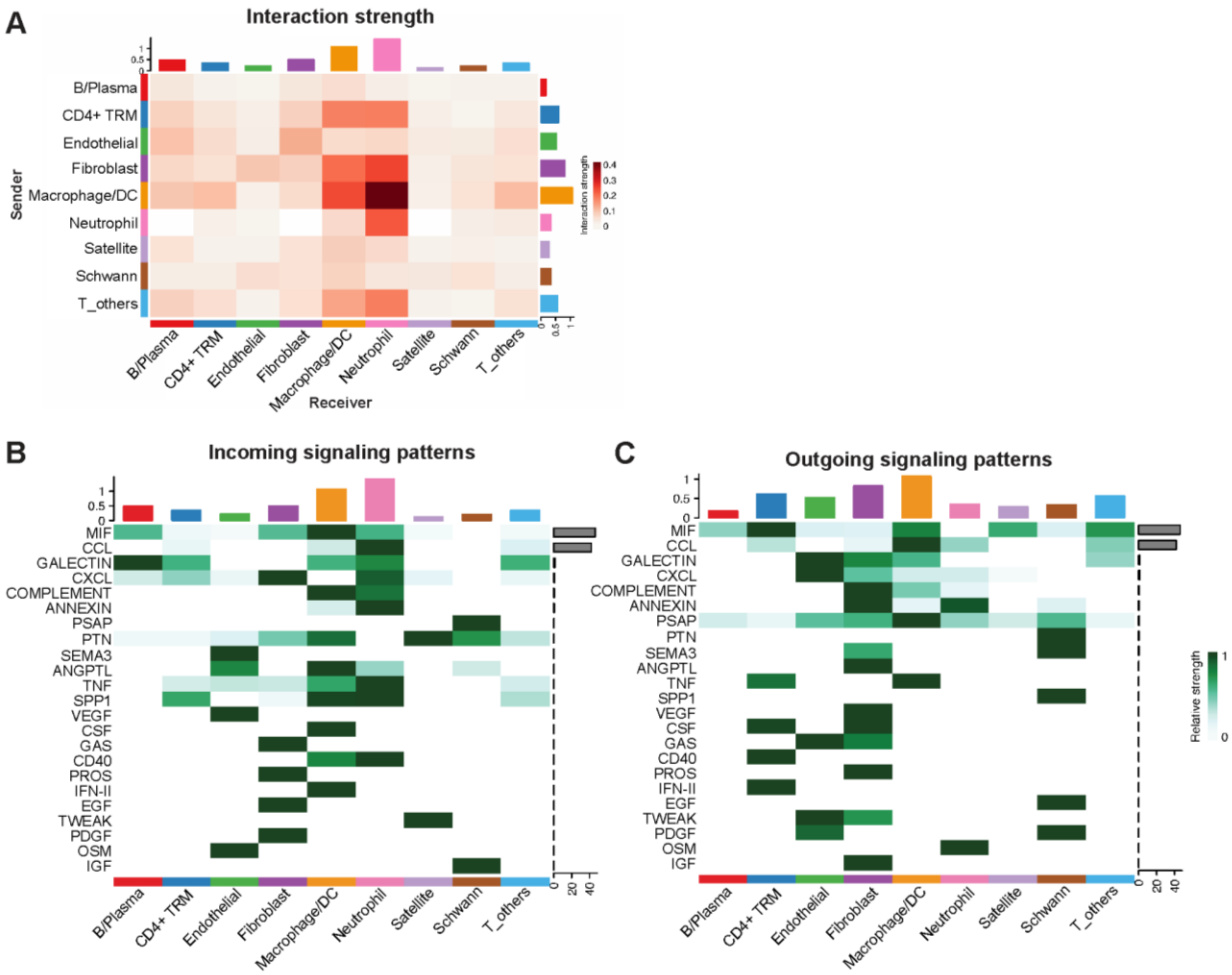

Supplementary Figure 9. Cell-cell communication among CD4+ TRMs and other clusters. (A) Interaction strength between CD4+ TRMs and other clusters, visualized by the depicted heatmap. Interaction strength represents ligand-receptor mediated intercellular communication probability, measured by CellChat. Horizontal and longitudinal axes indicate incoming and outgoing signals, respectively. Vertex size is proportional to cell counts in each cluster. (B) Incoming signaling patterns across each cluster visualized by heatmap. (C) Outgoing signaling patterns across each cluster visualized via heatmap.

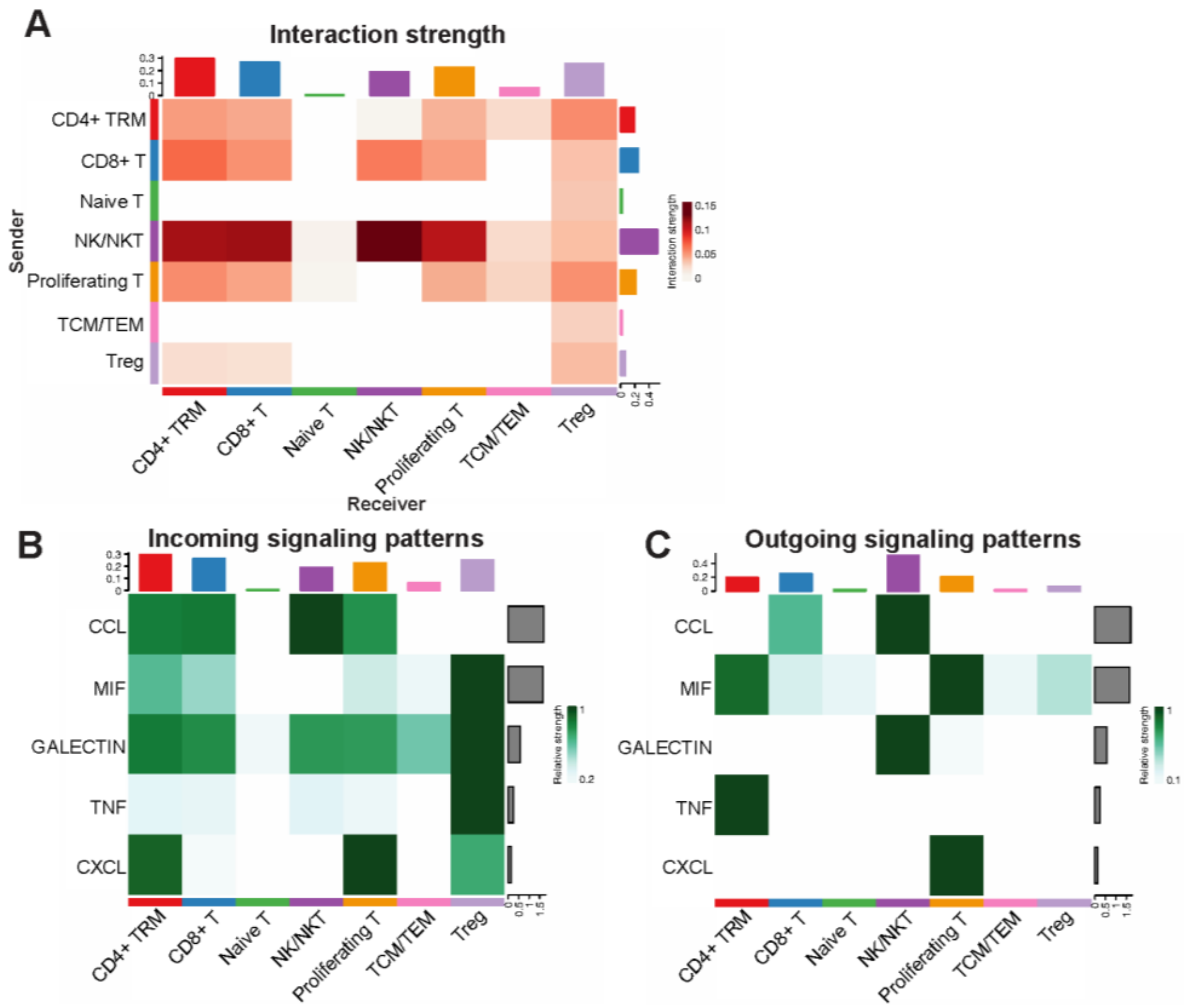

Supplementary Figure 10. Cell-cell communication among CD4+ TRMs and other subsets of T/NK cells. (A) Interaction strength between subsets of T/NK cells, depicted by heatmap. Interaction strength represents ligand-receptor mediated intercellular communication probability, measured by CellChat. Horizontal and longitudinal axes indicate incoming and outgoing signals, respectively. Vertex size is proportional to cell counts in each cluster. (B) Incoming signaling patterns across each subset of T/NK cells visualized by heatmap. (C) Outgoing signaling patterns across each subset of T/NK cells visualized via heatmap.

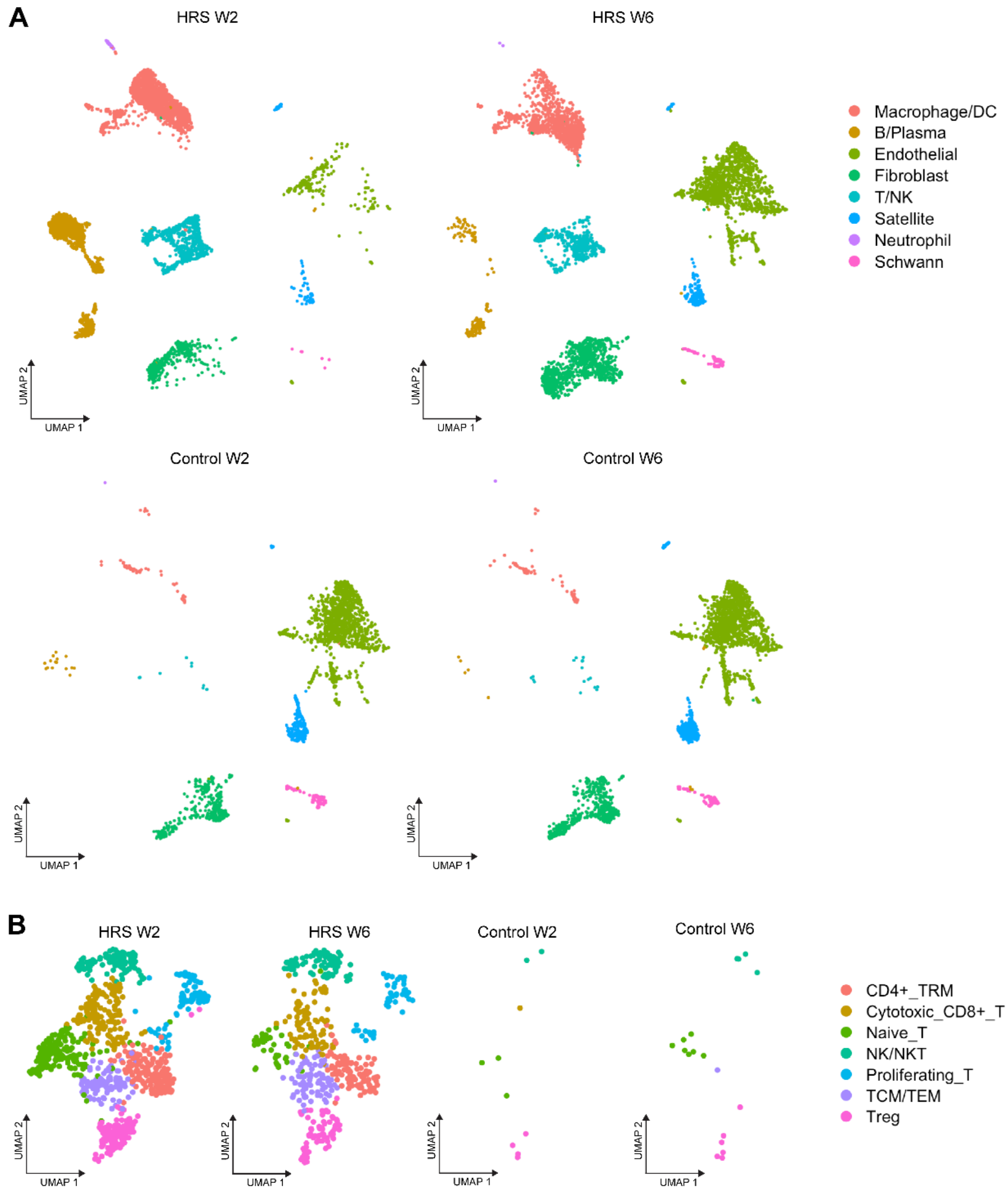

Supplementary Figure 11. Muscle infiltrates 2 vs 6 weeks post HRS immunization. (A) Muscle infiltrates from HRS-immunized and control groups were visualized via UMAPs corresponding to 2- and 6-week time points post HRS immunization. (B) T/NK subclusters from HRS-immunized and control groups were visualized via UMAPs and divided by 2- and 6-week time points post immunization.

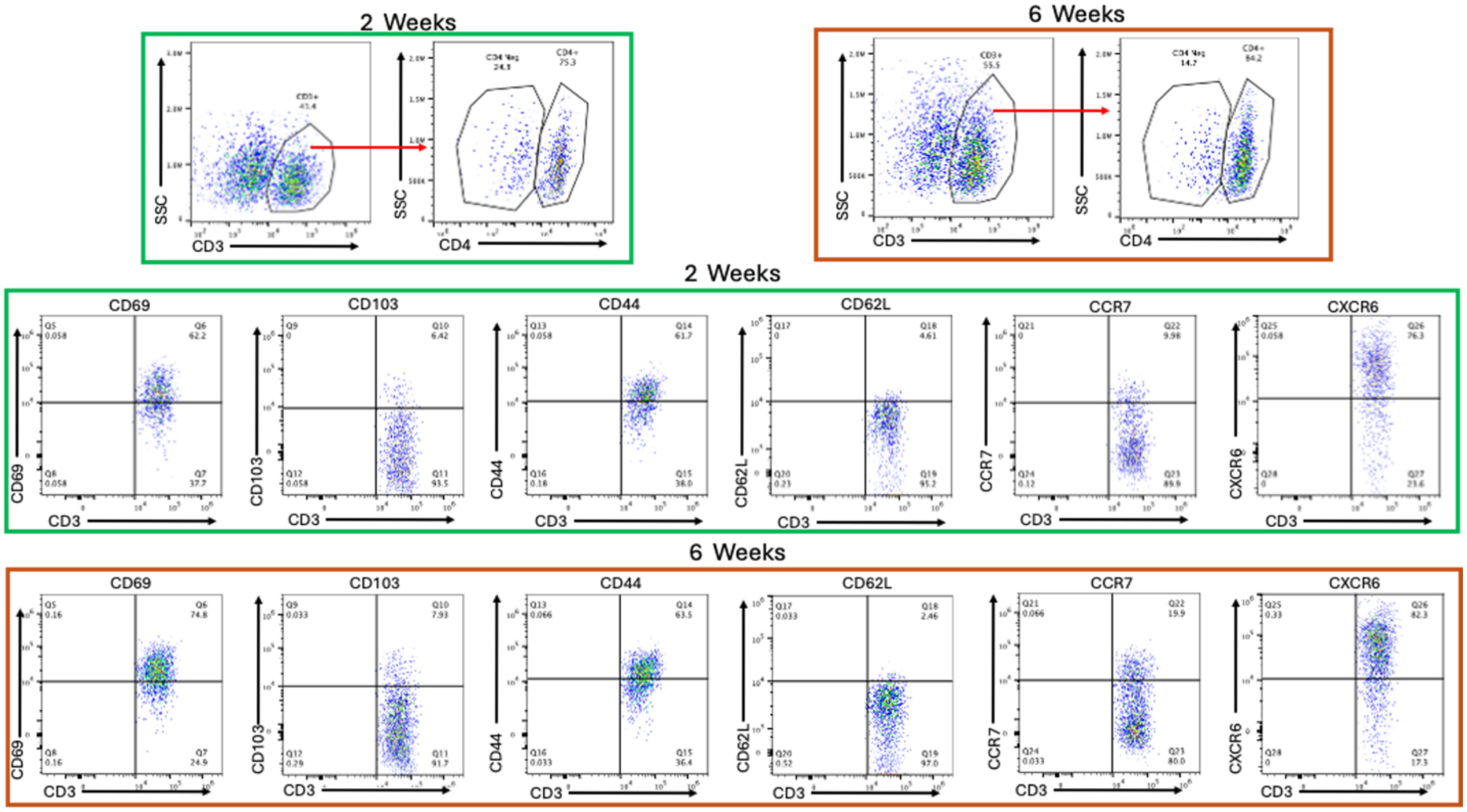

Supplementary Figure 12. Stability of the CD4+ TRM phenotype over time. Spectral flow cytometry was used to assess stability of the CD4+ TRM phenotype at 2 weeks and 6 weeks post-intramuscular injection with recombinant HRS. CD4+ TRM-like cells were CD69+, CD103^{low}, CD44^{high}, CD62L^{low}, CCR7^{low}, and CXCR6^{high}.

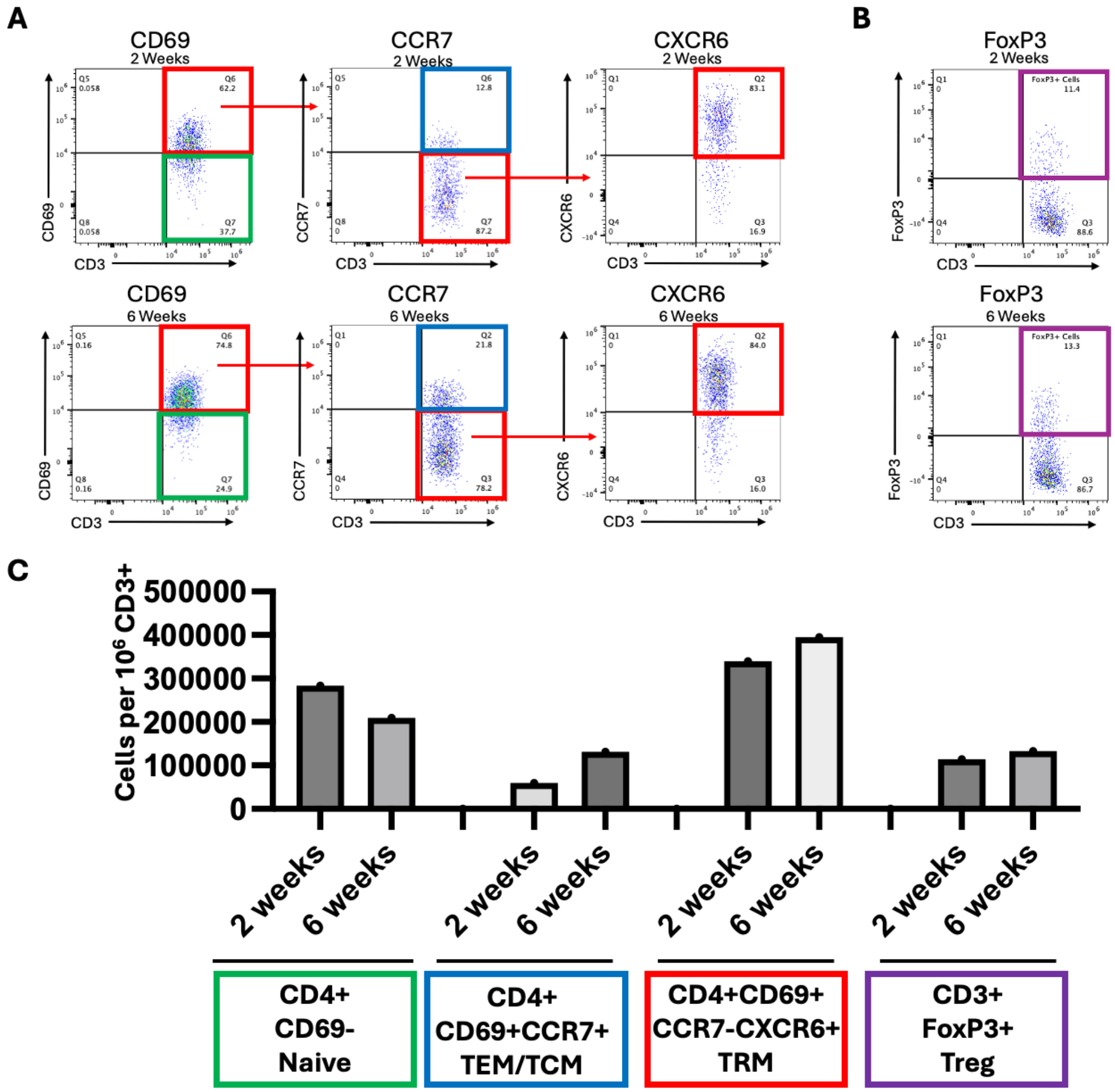

Supplementary Figure 13. Proportions of CD4+ cell subpopulations over time following intramuscular injection of recombinant HRS. (A) CD4+ cells were subphenotyped by spectral flow cytometry to identify CD4+CD69- Naïve T cells (green), CD4+CD69+CCR7+ T effector memory/T central memory (TEM/TCM) cells (blue), and CD4+CD69+CCR7-CXCR6+ TRMs (red). (B) A similar approach was used to identify total CD3+FoxP3+ Tregs (purple). Based upon percentages of cells obtained in (A) and (B), the number of each cell population per 1 X 10^6 viable CD3+ T cells was calculated (C). While 2 week samples were pooled from n=2 HRS-immunized mice, 6 week samples were comprised of muscle-infiltrating lymphocytes from n=3 mice.

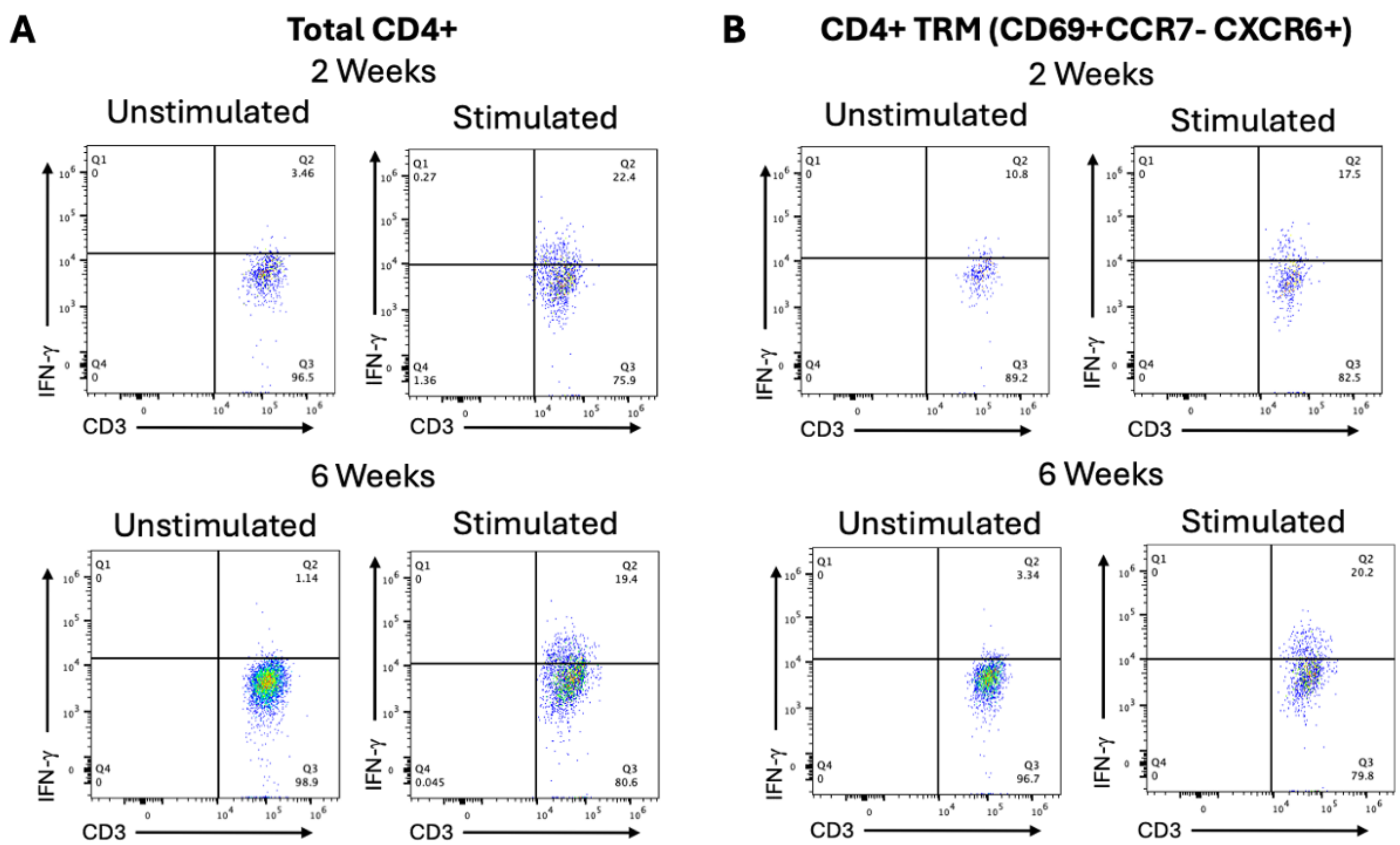

Supplementary Figure 14. IFN-γ expression in muscle CD4+ TRMs. Unstimulated and stimulated (T cell activation beads; PMA plus Ionomycin) muscle-infiltrating cells were assessed for IFN-γ expression by flow cytometry 2 and 6 weeks post-immunization with recombinant HRS. Shown are results for (A) total CD4+ cells, and (B) CD69+CCR7-CXCR6+ CD4+ TRMs.
